# Structural Elucidation of the Mechanism of Molecular Recognition in Chiral Crystalline Sponges

**DOI:** 10.1002/anie.202006438

**Published:** 2020-08-11

**Authors:** Shi‐Yuan Zhang, David Fairen‐Jimenez, Michael J. Zaworotko

**Affiliations:** ^1^ Department of Chemical Science Bernal Institution University of Limerick Limerick V94 T9PX Republic of Ireland; ^2^ Department of Chemical Engineering & Biotechnology University of Cambridge Cambridge CB3 0AS UK

**Keywords:** chiral binding sites, chiral crystalline sponge, chiral metal–organic frameworks, chiral separation and resolution, molecular recognition

## Abstract

To gain insight into chiral recognition in porous materials we have prepared a family of fourth generation chiral metal–organic frameworks (MOFs) that have rigid frameworks and adaptable (flexible) pores. The previously reported parent material, [Co_2_(S‐mandelate)_2_(4,4′‐bipyridine)_3_](NO_3_)_2_, CMOM‐**1S**, is a modular MOF; five new variants in which counterions (BF_4_^−^, CMOM‐**2S**) or mandelate ligands are substituted (2‐Cl, CMOM‐**11R**; 3‐Cl, CMOM‐**21R**; 4‐Cl, CMOM‐**31R**; 4‐CH_3_, CMOM‐**41R**) and the existing CF_3_SO_3_^−^ variant CMOM‐**3S** are studied herein. Fine‐tuning of pore size, shape, and chemistry afforded a series of distinct host–guest binding sites with variable chiral separation properties with respect to three structural isomers of phenylpropanol. Structural analysis of the resulting crystalline sponge phases revealed that host–guest interactions, guest–guest interactions, and pore adaptability collectively determine chiral discrimination.

## Graphical abstract

The chiral recognition mechanism of a family of chiral metal–organic materials was investigated towards the resolution of three racemic mixtures of phenylpropanol. X‐ray single‐crystal analysis of host–guest interactions revealed the specific binding sites with shape complementarity between the guest molecules and the adaptable chiral cavity.

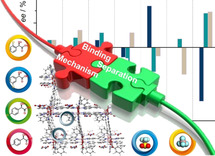

## Introduction

The existence of chirality in biology makes the production of pure enantiomers important to the manufacture of pharmaceuticals, agrochemicals, flavorings, and fragrances. The development of new techniques and materials for asymmetric synthesis and enantiomeric separation has therefore received great attention. For example, whereas some enantiomers of a drug molecule have no activity, some can have different toxicology, being harmful or toxic.^1^ Asymmetric synthesis is an elegant solution when available, but many times it is more economical to synthesize molecules as racemic mixtures and then separate the enantiomers. In this context, polysaccharide‐ and cyclodextrin‐based chiral stationary phases (CSPs) have been extensively used for chiral chromatography with efficacy determined by the stereospecificity of surfaces and cavities.^2^ However, the chiral recognition mechanisms in these materials are not well understood, especially across a range of chiral compound types.

Metal–organic materials (MOMs)^3^ are crystalline materials comprised of metal ions or clusters and organic ligands, making them modular and capable of exhibiting extra‐large porosity. In particular, chiral MOMs (CMOMs) can be designed to combine homochirality and porosity such that, when the pore size shows a good match for the targeted guest, they can provide a well‐defined stereospecific environment for the tight fit that is required for the discrimination and separation of enantiomers.^4^ Previously, racemic alcohols, ketones, amines, amides, acids, sulfoxides, and diols have been examined in terms of enantioselectivity by CMOM adsorbents, CSPs, and membranes.^4^, ^5^ Other types of porous solids investigated in this context include hydrogen‐bonded frameworks,^6^ covalent organic frameworks (COFs),^7^ and porous organic cages.^8^ Although more traditional porous materials such as zeolites have also been studied, the relative instability of the homochiral network^9^ limits their potential. Thus far, few porous materials have been studied to specifically address the origin of enantioselectivity^5^, ^6^, ^10^ and the nature of the interactions that promote enantioselective separation remains understudied. This is mainly because guest disorder, partial occupancy and high symmetry of the host can preclude accurate structural determination at the molecular level.

Herein, we address the mechanism of chiral recognition in a family of CMOMs derived from the parent structure [Co_2_(man)_2_(bpy)_3_](NO_3_)_2_ (man=*S*‐mandelate, CMOM‐**1S**, or *R*‐mandelate, CMOM‐**1R**, bpy=4,4′‐bipyridine).5b X‐ray crystallography can be a powerful tool in this context as it can provide in situ information about the supramolecular interactions that drive host‐guest binding. To our knowledge, CMOM‐**3S** is the only CMOM that can serve as both a general‐purpose crystalline sponge^11^ and a chiral stationary phase for enantioselective separation/identification of racemic mixtures.^12^ We have therefore exploited the modular nature of CMOM‐**1S/R**, for which both the counterion and mandelate linker are amenable to substitution. The resulting family of CMOMs detailed herein exhibits a hard rigid framework and adaptable or soft pores.^13^ These materials can therefore be classified as a fourth‐generation MOFs.^14^ The parent CMOM, CMOM‐**1S**, can be readily fine‐tuned through substitution of the counterion (BF_4_^−^, CMOM‐**2S**; CF_3_SO_3_^−^, CMOM‐**3S**) or linker ligand (2‐Cl, CMOM‐**11R**; 3‐Cl, CMOM‐**21R**; 4‐Cl, CMOM‐**31R**; 4‐CH_3_, CMOM‐**41R**). As detailed below, this family of CMOMs can serve as chiral crystalline sponges (CCSs) to provide structural insight into the supramolecular interactions that occur between phenylpropanols and the pore surface of this family of CMOMs.

## Results and Discussion

**Figure 1:**
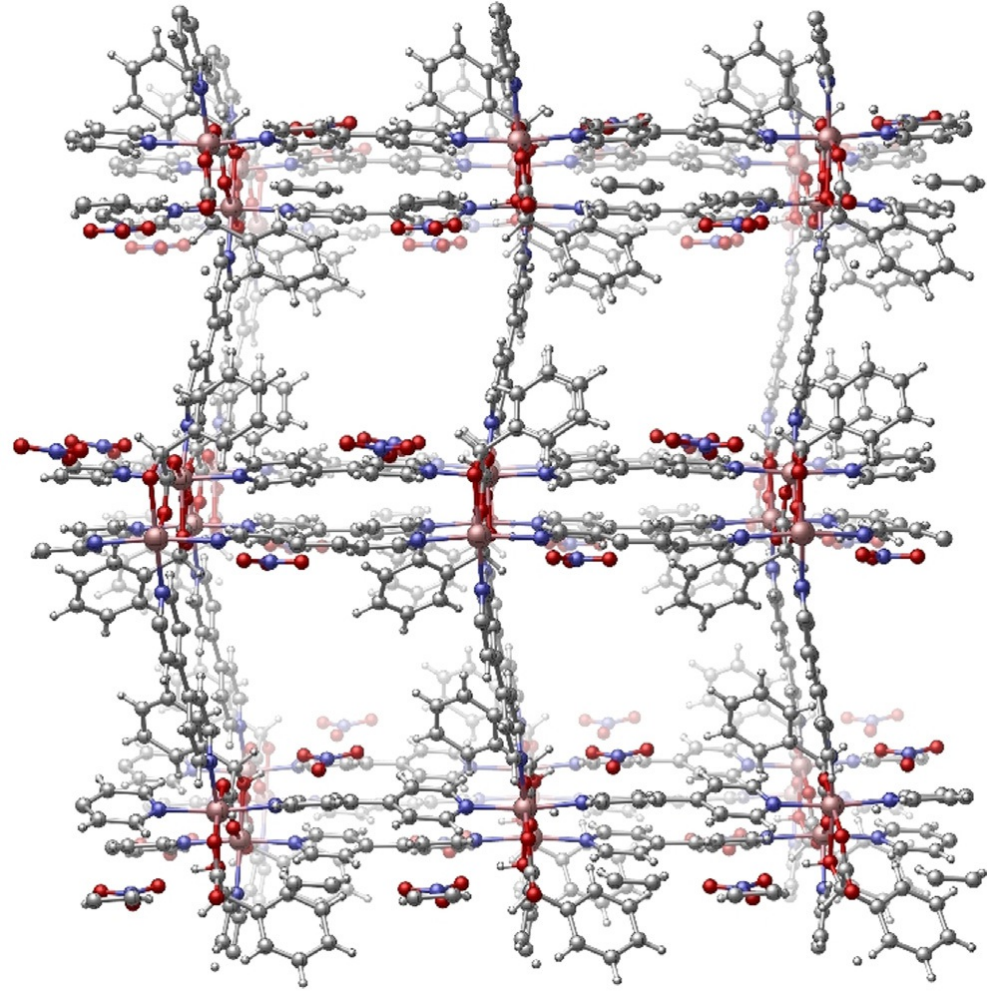
Crystalline structure of the parent material, CMOM‐**1S**, from where the seven isostructural CMOMs are derived, all showing **bnn** topology. Solvent molecules have been removed for clarity. Table 1 lists the structural components of the seven CMOMs.

CMOM‐**1S/R** is comprised of inexpensive, commercially available ligands and its modular nature enables the metal ions, linkers and anions to be substituted. Figure 1 shows the crystal structure of this CMOM whereas Table 1 lists their structural components. In principle, this modularity should enable the generation of a diverse platform of isostructural derivatives for the systematic study of the factors that influence chiral discrimination in CMOMs.

**Table 1:** Structural components of the seven CMOMs studied herein.

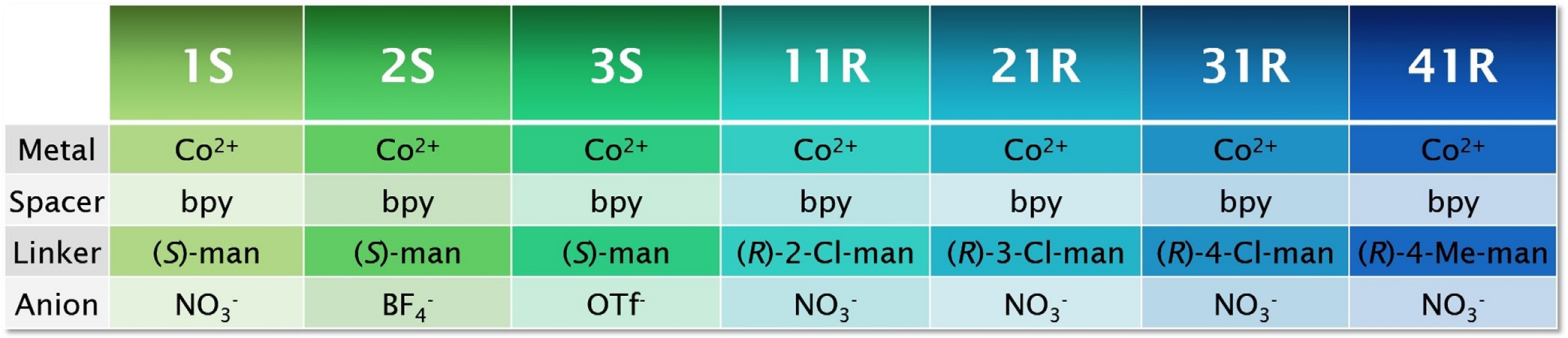

### Structural Features

Based on the parent structure (Figure 1), six variants of **1S** and **1R** were prepared in which we either replaced nitrate anions with tetrafluoroborate (BF_4_^−^) or triflate (OTf^−^) anions or incorporated Cl/CH_3_‐substituted mandelate anions as ligands (Table 1). In the case of anion variation, we named the samples according to their composition as [Co_2_(*S*‐man)_2_(bpy)_3_](BF_4_)_2_ (**2S**); [Co_2_(*S*‐man)_2_(bpy)_3_](OTf)_2_ (**3S**); in the case of the ligand substitution, we named the samples as [Co_2_(*R*‐2‐Cl‐man)_2_(bpy)_3_](NO_3_)_2_ (**11R**); [Co_2_(*R*‐3‐Cl‐man)_2_(bpy)_3_](NO_3_)_2_ (**21R**); [Co_2_(*R*‐4‐Cl‐man)_2_(bpy)_3_](NO_3_) (**31R**); [Co_2_(*R*‐4‐CH_3_‐man)_2_(bpy)_3_](NO_3_)_2_ (**41R**). We obtained crystals of each CMOM by solvent diffusion between MeOH solutions of the appropriate cobalt(II) salt and enantiopure mandelic acid layered over aromatic solvent solutions of bpy. Single crystal X‐ray diffraction analysis revealed that these CMOMs crystallize in chiral space group *P*2_1_. All six variants exhibit **bnn** topology and are isostructural with **1S**/**1R**, that is, they are comprised of rod building blocks based upon *S/R*‐man, bpy ligands and extra‐framework anions. Pore chemistry, size and shape are defined by the mandelate substituents and the position of the counterions. The maximum aperture of the 1D channels in these CMOMs is fixed because it is controlled by the length of the bpy linkers, resulting in ca. 0.8×0.8 nm pore diameter (taking into account the van der Waals radii). The void volume was calculated to be 33 % (**1S**), 28 % (**2S**), 25 % (**3S**), and 31 % (**11R**, **21R**, **31R**, and **41R**) of the unit cell volume. The size of the anions has a greater impact on the pore volume than the functionality of the mandelate ligand. The powder X‐ray diffraction (PXRD) patterns of as‐synthesized CMOMs (Supporting Information, Figures S1–S6) closely match those calculated from single‐crystal data. PXRD was also used to verify the structural integrity of CMOMs after solvent exchange and guest molecule loading. Thermogravimetric analysis revealed 10–20 % weight loss upon initial heating (Supporting Information, Figure S7), related to solvent loss, and thermal stability of 200–300 °C.

### Chiral Resolution of Phenylpropanols

**Scheme 1:**
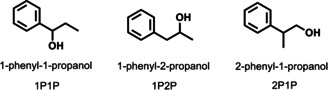
Structures of the three phenylpropanols used for chiral discrimination studies.

We selected three structural isomers of phenylpropanol (Scheme 1), namely 1‐phenyl‐1‐propanol (1P1P), 1‐phenyl‐2‐propanol (1P2P) and 2‐phenyl‐1‐propanol (2P1P) to investigate the chiral discrimination of our CMOM family towards their racemic mixtures. Enantiopure phenylpropanols are important intermediates used in the synthesis of pharmaceutical and parasiticide compounds.^16^ Chiral resolution experiments were conducted using our previously reported procedure5b on activated CMOM crystals (see the Experimental Section in the Supporting Information for full details). The activated crystals were soaked in racemic mixtures of phenylpropanols for 5 days before the crystalline solids were filtered and washed with cyclohexane to remove the excess of molecules adsorbed on the external surface. We then extracted the encapsulated phenylpropanols from the crystals using dichloromethane and we evaluated the enantioselective separation performance by analyzing the composition of the eluate through HPLC.

**Figure 2:**
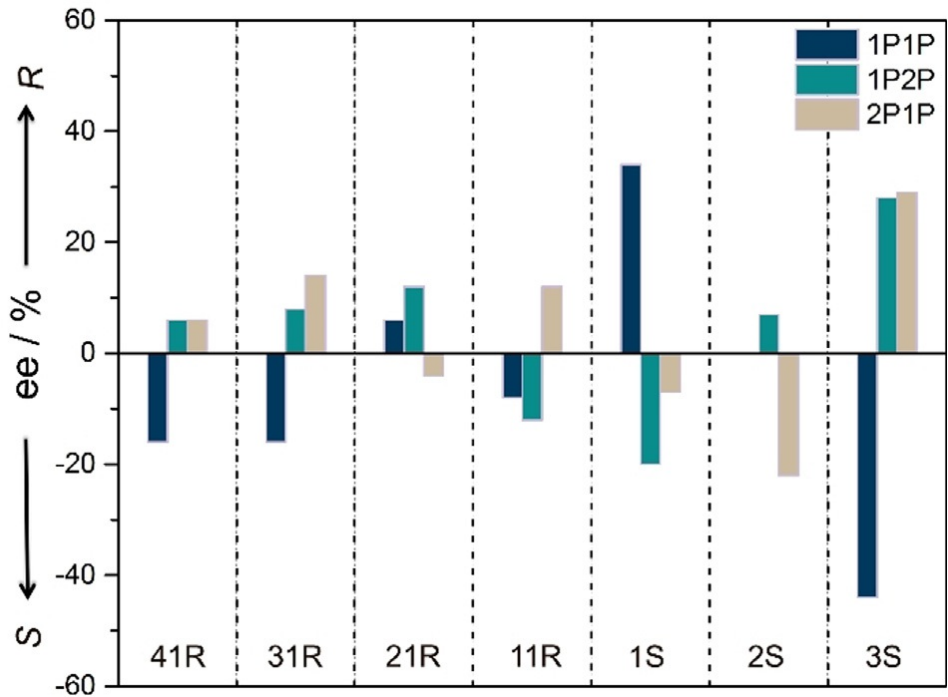
Chiral resolution of phenylpropanol (1P1P, 1P2P, and 2P1P) racemic mixtures after contact by CMOMs at room temperature for 5 days. Enantiomeric excess (*ee*) values were determined by HPLC analysis using chiral IB and ID stationary phase.

Figure 2 shows the results of the chiral separation of 1P1P, 1P2P, and 2P1P by CMOM‐**1S**, **2S**, **3S**, **11R**, **21R**, **31R**, and **41R**. Interestingly, the outcomes differ in both enantiomeric excess (i.e., separation performance) and absolute values. For example, whereas we observed a 31 % *ee* resolution of (*R*)‐1P1P for **1S** (66 % *ee* based on a different resolution method),5b we observed no separation and opposite enantioselectivity for **2S** and **3S** structures, with 0 % and −42 % *ee*, respectively. As the cationic framework for **1S**, **2S**, and **3S** is the same in terms of structure and chirality, these differences in performance must be attributed to the EFA (i.e., NO_3_^−^, BF_4_^−^, CF_3_SO_3_^−^, respectively) and its impact upon pore shape and surface chemistry. Conversely, when we changed the functionality of the mandelate ligand, we observed a lower *ee* (<20 %) for **11R** (2‐Cl‐man), **21R** (3‐Cl‐man), **31R** (4‐Cl‐man), and **41R** (4‐Me‐man) vs. that observed for **1S**. Furthermore, **21R**, preferentially bonded to the opposite enantiomer preferred by **11R**, **21R**, **31R**, and **41R**. These results indicate that even subtle structural effects can strongly impact chiral discrimination in these CMOMs.

Looking at the three regioisomers of phenylpropanol (Scheme 1), we found that the difference in the position of the hydroxyl group strongly affects the enantioselectivity. In the case of **1S**, (*R*)‐1P1P was preferentially adsorbed, whereas it favored the adsorption of (*S*)‐1P2P and (*S*)‐2P1P. A similar phenomenon was observed for **3S** but with the opposite binding selectivity (up to 42 % *ee*) for these three phenylpropanols. The highest degree of separation was achieved by **3S** for 1P2P and 2P1P. Our results should be placed in the following context: the most widely used method of assigning the relative and absolute configurations of a series of related compounds is through asymmetric synthesis or chiral separation. Typically in asymmetric synthesis, the structure of one of a group of new compounds is determined by single‐crystal X‐ray diffraction (SCXD), and, by analogy, the same configuration is assigned to related compounds. The reverse enantioselectivity observed herein suggests that the assignment of chirality by analogy is not reliable.

### Chiral Recognition Mechanism Studies

**Figure 3:**
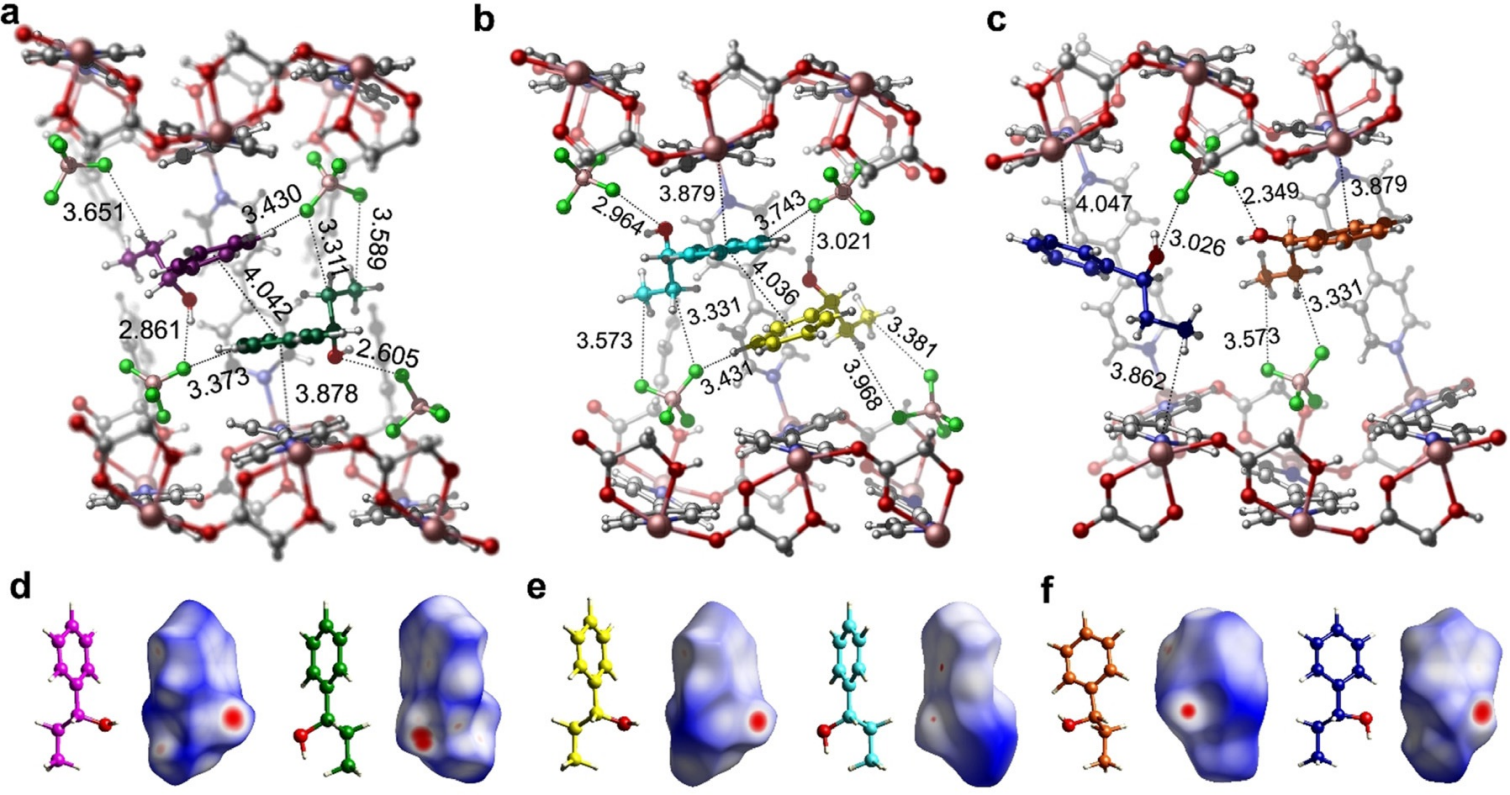
The six guest binding sites of 1P1P in CMOM‐**2S** as determined by SCXRD. Location of six crystallographically independent 1P1P molecules (colored magenta, green, yellow, light blue, orange, and dark blue) in the chiral channel of CMOM‐**2S** with interactions of the first (a), second (b), and third (c) enantiomeric pairs of 1P1P molecules within the cavity. Absolute configuration and Hirshfeld surface of the six independent 1P1P molecules (d–f).

Perhaps the most salient aspect of the results reported above is the variable enantioselectivity that occurs from subtle changes in the composition of the CMOMs. To better understand these results we determined the nature of the host–guest binding sites from SCXRD studies of the guest loaded CMOM crystals. The observation of 0 % *ee* by 1P1P‐loaded **2S** stands out as being anomalous. Intuitively, one would expect that all chiral porous materials will exhibit at least some degree of chiral discrimination unless size exclusion happens. Indeed, whereas previous studies on homochiral hosts such as CMOMs, COFs and metal–organic cages have revealed examples of chiral materials with low or moderate enantioselectivity, to the best of our knowledge, 0 % *ee* has not yet been reported.^5^, ^17^ Thanks to the crystalline sponge nature of this class of CMOMs we are in position to elucidate the nature of the intermolecular interactions that resulted in 0 % *ee* in **2S**, a chiral porous material. The structure of the 1P1P‐loaded **2S** reveals that the unit cell is doubled vs. as‐synthesized **2S** along *b* axis, and that there are six crystallographically independent 1P1P molecules in the structure. As shown in Figure 3, the orientation between two enantiomeric pairs of 1P1P molecules is perpendicular, presumably to maximize the packing efficiency and guest interactions with the framework. In the first pair of 1P1P molecules, there are π–π interactions between them and two bpy ligands of the framework (Figure 3 a). In addition, there are hydrogen bonding interactions between two 1P1P molecules and four surrounding BF_4_^−^ counterions, with C_π_−H⋅⋅⋅F, C_alkyl_−H⋅⋅⋅F, and O−H⋅⋅⋅F interactions ranging from 2.605 to 3.651 Å. The packing of the second pair of 1P1P molecules is similar to that of the first pair, but offsetting of two phenyl rings results in a directional C_alkyl_−H⋅⋅⋅π interaction between two 1P1P molecules (Figure 3 b). In the third pair of 1P1P molecules, both phenyl rings of 1P1P exhibit close contacts with pyridyl moieties from the framework. Both hydroxyl groups interact with the same BF_4_^−^ ion through hydrogen bonds. Hirshfeld surface analysis^18^ of 1P1P molecules reveals that they are tightly encapsulated in the chiral channel (Figure 3 d–f). Notably, each enantiomeric pair contains equal amounts of (*S*)‐ and (*R*)‐1P1P, leading to a 0 % *ee* from crystallographic analysis, which is fully consistent with the experimentally measured discrimination results.

**Figure 4:**
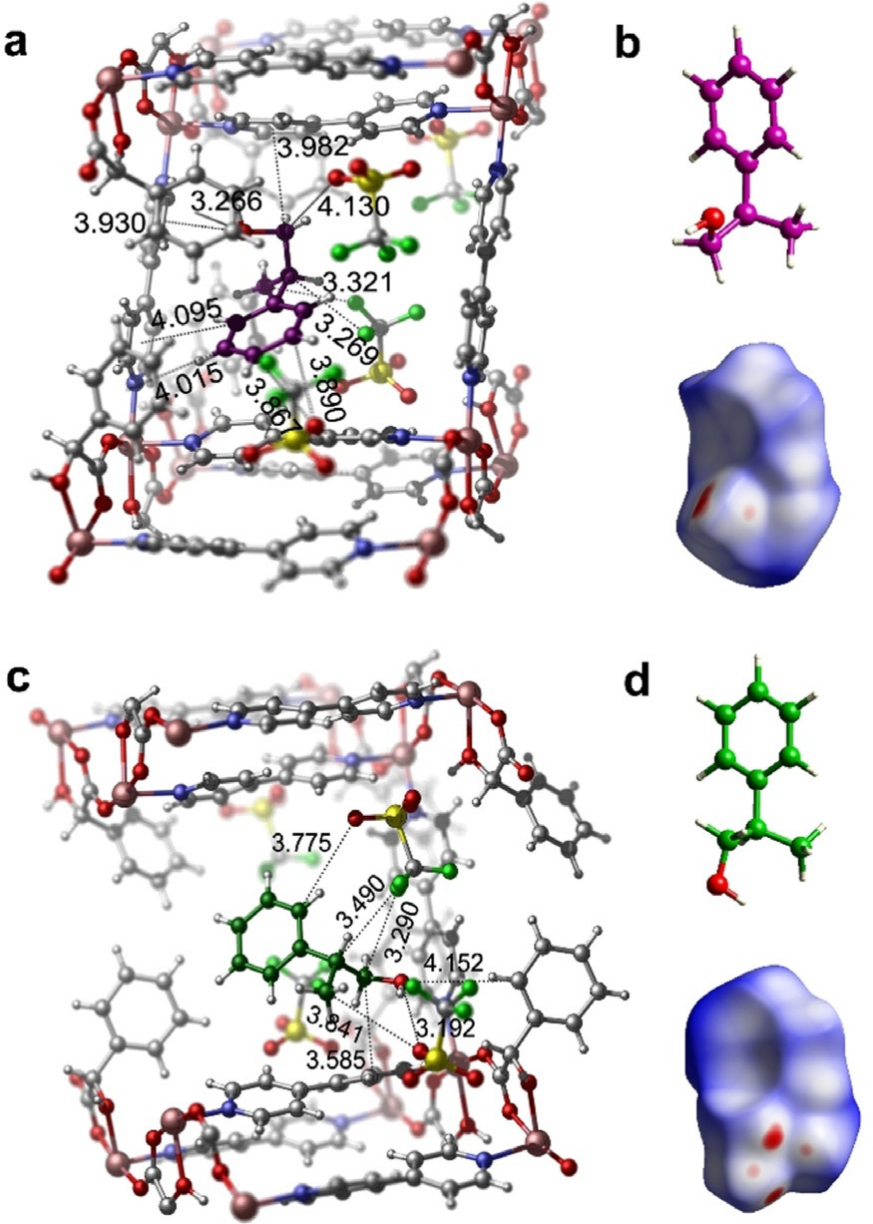
Guest binding sites of 2P1P in CMOM‐**3S** as determined by SCXRD. Interactions of position disordered 2P1P molecules (colored magenta and green) in their respective cavities (a and c). Absolute configuration and corresponding Hirshfeld surface of 2P1P molecules (b and d).

To better understand the variability of the absolute configuration of guests within the same scaffold, we determined the structures of **3S** loaded with each of the three phenyl propanols. The host‐guest binding sites for 1P1P and 1P2P were discussed in our earlier paper.^12^ Intermolecular π‐π, C−H⋅⋅⋅π, and hydrogen bonding interactions contribute to the effective enantioselective recognition of (*S*)‐1P1P and (*R*)‐1P2P. In the case of 2P1P, we did not observe π–π interactions between 2P1P and bpy ligands (Figure 4 a). Instead, the phenyl ring of 2P1P forms hydrogen bonds and π‐interactions through C_π_−H⋅⋅⋅X (X=O, F and π). Hydrogen bonding and C_alkyl_−H⋅⋅⋅π interactions between the side chain of 2P1P and the framework dictates the orientation of 2P1P. One disordered 2P1P is located in a different binding site, wherein the terminal propyl alcohol interacts with triflate anions through C_alkyl_−H⋅⋅⋅X (X=O, F) and O−H⋅⋅⋅O hydrogen bonds (Figure 4 b). The strength and number of interactions within the chiral channel transform the binding sites for (*R*)‐2P1P. The Hirshfeld surface analysis of 2P1P reflects once more the close contacts detailed above.

### The Role of the EFAs on the Mechanism of Molecular Recognition

**Figure 5:**
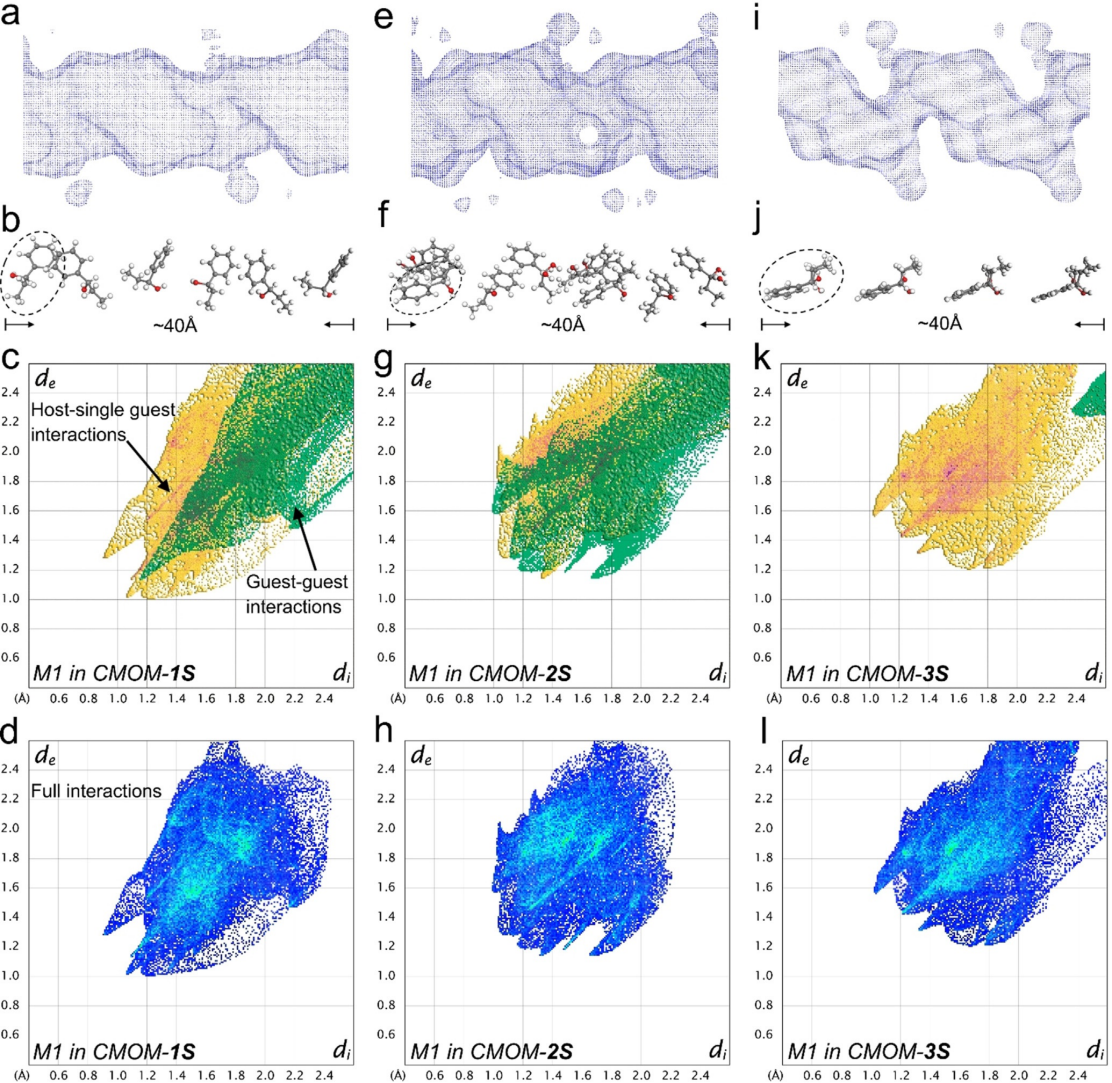
X‐ray crystallographic analysis of the guest binding of 1P1P in CMOM‐**1S** (left column), **2S** (middle column), and **3S** (right column). The shape and size of the channel of **1S** (a), **2S** (e), **3S** (i) containing 1P1P guests is visualized as a Connolly surface generated with probe size 1.2 Å. The 1P1P guest molecules in the channel **1S** (b), **2S** (f), **3S** (j) with distinctive orientation and alignment are shown with a length of around 40 Å. The partial (c, g, and k) and full (d, h, and l) molecular interactions of 1P1P molecule highlighted in dash circle (M1) are presented as 2D fingerprint plots. In these plots, yellow areas indicate the interactions with host framework and anions while the green area indicates the interactions with the rest of guest molecules. Blue color was used in full interaction maps. The intense and highlight area (red and cyan color) represents the greatest contribution to surface.

We focused then on the analysis of the mechanism of enantiomeric separation working with three structures with different anions (**1S**, **2S**, and **3S**) and one racemic mixture, 1P1P. The increasing size of anions used in the chiral channel of **1S**, **2S**, and **3S**, that is, NO_3_^−^, BF_4_^−^ to CF_3_SO_3_^−^, respectively, results in decreasing pore volume from **1S** to **3S**; Figure 5 shows these differences. Looking at the adsorption of 1P1P, whereas the cross‐section of the largest pore cavity of the structures is similar (8×8 Å), it is the pore shape and surface chemistry what defines the interactions with the 1P1P guest molecules. In **2S**, the guest molecules pack with higher density than those of **1S** (Figure 3 and Figure 5), while the 1P1P molecules in **3S** were isolated between two junctions with the distance of 10.2 Å. To analyze the supramolecular interactions of each guest molecule, we performed the fingerprint plots to highlight specific close‐contacts from host‐single guest and guest‐guest contributions; Figure 5 c–i and the Supporting Information, Figures S9–S11 show the full interaction. The interaction map was constructed by defining distances from the Hirshfeld surface to the nearest nucleus inside the surface (or internal, *d*_i_) and outside the surface (or external, *d*_e_) as the first functions of distance explored for mapping on the surfaces. The visual comparison between these three plots in terms of area in Figure 5 c, g, and k demonstrates the significant contribution of guest–guest interactions in **2S** that arise from π–π and C−H⋅⋅⋅π interactions. We note that the shape of guest‐guest interactions in **2S** is spread widely over the range of *d*e, *d*i <1.8 Å, rather than the narrow needle‐shape found in 1S, indicating the substantially close contacts among the neighboring guests in **2S**. Besides, the 1P1P molecules in the three structures shown in Figure 5 all display strong interactions with the host structures, as evidenced by the highlighted area colored with red and cyan. From the above analysis, one can infer that the most strongly bound of two enantiomers within the host will hinder chiral discrimination ability.

### The Impact of Guest Geometry on the Mechanism of Molecular Recognition

**Figure 6:**
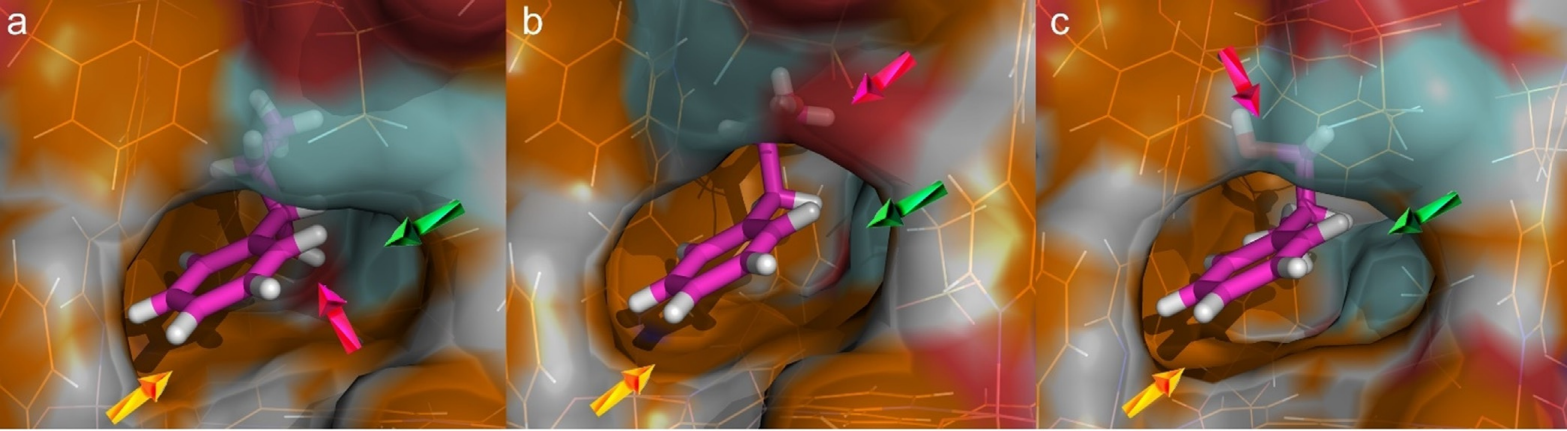
X‐ray structural analysis study of the guest binding pockets of 1P1P (a), 1P2P (b), and 2P1P (c) in CMOM‐**3S**. CMOM‐**3S** discriminates between similar substrates. The color of the Connolly surface represents the element that generates the corresponding part of the surface: C orange, O red, N blue, F cyan, H white. The carbon atoms of the substrates are colored magenta. The yellow, green, and red arrows indicate the aromatic, hydrophobic, and hydrophilic surface of the CMOM, respectively.

Following the analysis on the impact of the anion, on the enantiomeric separation, we compared the separation of the three molecules, 1P1P, 1P2P, and 2P1P, by **3S**. Looking across the three guest molecules loaded in the **3S** structures, their positions within the chiral channels are consistent, with one binding site being common to all three PPs. Figure 6 illustrates this idea; the phenyl rings of the PP molecules interact with the aromatic surface (orange) created by the phenyl and pyridyl rings of the framework, whereas the hydrophobic surface generated by the −CF_3_ moieties repel the −OH moieties and attracts the alkyl C−H groups. Here, the hydroxy groups of the PPs are stabilized by the hydrophilic surface of the framework through hydrogen bonding interactions taking place in different positions, as indicated by the red arrows in Figure 6. While the position and inclination of the head of the PP phenyl rings remain broadly the same, the orientation of the tail varies as a result of weak interactions. The specific chemical environment provided by the host appears to mimic enzyme binding sites in terms of the tight fit and ability to discriminate between enantiomers.^19^ Unfortunately, our attempts to accomplish the X‐ray analysis of the guest‐included crystals of CMOMs **11R**‐**41R** failed due to the poor crystallinity of these systems. This is not completely unexpected and it is very likely linked to the fact that these CMOMs show lower enantioselectivity. As such, the lack of distinct binding sites has a clear impact by the derivatization of the mandelate linker ligands.

## Conclusion

Modifying the pore chemistry of CMOMs as reported herein profoundly influences chiral discrimination properties. In each CMOM, adaptable pore size and shape resulted in tight binding sites that enable a variety of host—guest and guest–guest interactions. That the CMOMs can serve as crystalline sponges enabled the use of X‐ray crystallography to provide detailed analysis of short contacts at the molecular level and, in turn, provided insight into the molecular recognition phenomena that impact chiral separation. We have thereby demonstrated the feasibility of using a platform of CMOMs as crystalline sponges for systematic study of chiral discrimination in porous materials by manipulation of chiral pores while retaining the same framework structure. The resulting variability in enantioselectivity is quite dramatic considering the invariability of the cationic framework and means that this CMOM platform can be classified as a fourth‐generation MOF with hard–soft features that enable chiral discrimination and functioning as a crystalline sponge. That enantiomeric discrimination is driven by tight guest binding sites within the chiral cavity is likely to be a generally important feature of CMOMs that exhibit strong enantioselectivity. What is perhaps more important though is that the parent CMOM structure can be easily tuned to enable ad hoc enantiomeric separations. We foresee opportunities for the development of more sophisticated CMOMs with precisely controlled chiral environments that will open up a pathway for their use in stereospecific catalysis and for separation of enantiomers of biologically active compounds in the pharmaceutical industry.

## Conflict of interest

The authors declare no conflict of interest.

## Supporting information

As a service to our authors and readers, this journal provides supporting information supplied by the authors. Such materials are peer reviewed and may be re‐organized for online delivery, but are not copy‐edited or typeset. Technical support issues arising from supporting information (other than missing files) should be addressed to the authors.

Supplementary
