# Supplementary material for: Structural Elucidation of the Mechanism of Molecular Recognition in Chiral Crystalline Sponges
Source: Angew Chem Int Ed Engl. 2020 Aug 11;59(40):17600–6. doi: 10.1002/anie.202006438 (PMC7540565; doi:10.1002/anie.202006438)
Supplement: Supplementary file 1 — Supplementary [file ANIE-59-17600-s001.pdf]

Angewandte Chemie
Eine Zeitschrift der Gesellschaft Deutscher Chemiker

Supporting Information

Structural Elucidation of the Mechanism of Molecular Recognition in Chiral Crystalline Sponges

Shi-Yuan Zhang, David Fairen-Jimenez, and Michael J. Zaworotko**

anie_202006438_sm_miscellaneous_information.pdf

Table of Contents

Experimental Section	S2-S3
Hirshfeld surface analysis	S4
Characterization of CMOMs (Figures S1-S8)	S5-S12
Additional Figures (Figures S9-S11)	S13-S15
HPLC traces (Figures S12-S32)	S16-S36
Crystallographic data (Tables S1-S7)	S37-S43
Reference	S44

Experimental Section

1. Materials and Synthesis: All reagents and solvents were commercially available and used as received.

1.1. Synthesis of CMOM-**1S·NB** *and* CMOM-**3S·DCB**. The corresponding crystals were synthesized according to our published procedure[1,2].

1.2. Synthesis of CMOM-**2S·BTF**. A 5 mL methanol solution of 0.4 mmol $Co(BF_4)_2 \cdot 6H_2O$ (136 mg) and 0.4 mmol enantiopure (*S*)-mandelic acid (60.8mg) was layered above a 5 mL benzotrifluoride (BTF) solution of 0.3 mmol bpy (46.8 mg). The buffer solution of a 5mL 1:1 methanol/benzotrifluoride was layered between the top and the bottom layers to allow slow diffusion for 7 days. Red rectangular crystals were obtained in ~70% yield.

1.3. Synthesis of CMOM-**11R·NB**, **21R·NB**, **31R·NB**, *and* **41R·NB**. A general procedure was applied as **1S·NB**. (*R*)-2-chloromandelic acid (0.4 mmol, 740 mg), (*R*)-3-chloromandelic acid (0.4 mmol, 740 mg), (*R*)-4-chloromandelic acid (0.4 mmol, 740 mg), and (*R*)-4-methylmandelic acid (0.4 mmol, 660 mg) were used instead of (*S*)-mandelic acid for the synthesis of **11R·NB**, **21R·NB**, **31R·NB**, and **41R·NB**, respectively. Red rectangular crystals were isolated in ~40-50% yield.

1.4. Preparation of CMOMs Samples. Crystals of as-synthesized CMOMs were exchanged with DCM (5mL) for 5 times to successfully remove NB/DCB/BTF. Samples were stored in DCM for further usage.

2. Characterization.

2.1. Physical measurements: Powder X-Ray diffraction was performed on a PANalytical X'Pert MPD Pro using Cu Kα (λ = 1.5418 Å) radiation with a 1D X'Celerator strip detector. Thermogravimetric analysis was performed using a TA Instruments TGA-Q50 at a constant rate of 5ºC/min from 25ºC to 550ºC. HPLC measurements were carried out on a Shimadzu HPLC system with Chiralpak IB and ID columns (250 × 4.6 mm i.d.) with a flow rate of 1 mL/min.

2.2. Crystallographic studies: As-synthesized **2S·BTF**, **11R·NB**, **21R·NB**, **31R·NB**, **41R·NB** and guest exchanged **2S·1P1P**, **3S·2P1P** crystals are chosen for single crystal X-ray diffraction

study. The data were collected on a Bruker Quest PHOTON 100 CMOS system equipped with a Cu Kα INCOATEC Imus micro-focus source (λ = 1.54178 Å, T = 100(2) K). In all cases indexing was performed using APEX2.[3] Data integration and reduction were performed using SaintPlus 6.01.[4] Absorption correction was performed by multi-scan method implemented in SADABS.[5] Space groups were determined using XPREP implemented in APEX2. Structures were solved using Direct Method (SHELXS), expanded using Fourier methods and refined on F^2 using nonlinear least-squares techniques with SHELXL[6] contained in APEX2, WinGX v1.70.01[7] and OLEX2 v1.2.9[8] programs packages. Geometry calculations and checking for higher symmetry were performed with the PLATON program[9]. The SQUEEZE procedure within PLATON was applied for handling unordered solvent of **21R** and **31R**. Crystallographic data for the as-synthesized and guest exchanged CMOMs are summarized in Tables S1-S7.

3. Chiral Resolution of Phenyl Propanols (PPs).

The DCM exchanged CMOM crystals were evacuated under dynamic vacuum at room temperature. Desolvated materials were immersed in 0.5 mL racemic 1-phenyl-1-propanol (1P1P), 1-phenyl-2-propanol (1P2P), and 2-phenyl-1-propanol (2P1P) for 5 days without stirring or shaking, respectively. The crystallinity of the resulting PPs encapsulated CMOMs was examined by PXRD. Subsequently, CMOM crystals were filtered and washed with cyclohexane (6 × 1 mL) to remove the residual PPs from the surface of the crystals. DCM was then used to successively extract PPs from the crystals (8 × 0.5 mL). The resulting extracts were monitored by TLC to ensure that all encapsulated PPs had indeed been released. The filtrates were combined and analyzed by chiral HPLC to determine ee values. The resulting crystals were dried in air and weighed (weights ranged from 0.03~0.04 g).

Hirshfeld surface analysis

The Hirshfeld surface analysis was performed on CrystalExplorer.[10] Inside the Hirshfeld surface the electron distribution due to a sum of spherical atoms for the molecule (the promolecule) dominates the corresponding sum over the crystal (the procrystal), and the Hirshfeld surface is defined implicitly where the ratio of promolecule to procrystal electron densities equals 0.5. For each point on the surface, two colored distance properties are defined: d_e, the distance from the point to the nearest nucleus *external* to the surface, and d_i, the distance to the nearest nucleus *internal* to the surface. *Shape index* is a measure of "which shape", and it can be sensitive to very subtle changes in surface shape, particularly in areas where the total curvature (or the curvedness) is very low. 2D fingerprint plots are derived from the Hirshfeld surface by plotting the fraction of points on the surface as a function of the pair (d_i, d_e). Each point on the standard 2D graph represents a bin formed by discrete intervals of d_i and d_e (0.01 × 0.01 Å), and the points are colored as a function of the fraction of surface points in that bin, with a range from blue (relatively few points) through green (moderate fraction) to red (highest fraction).

Characterization of CMOMs

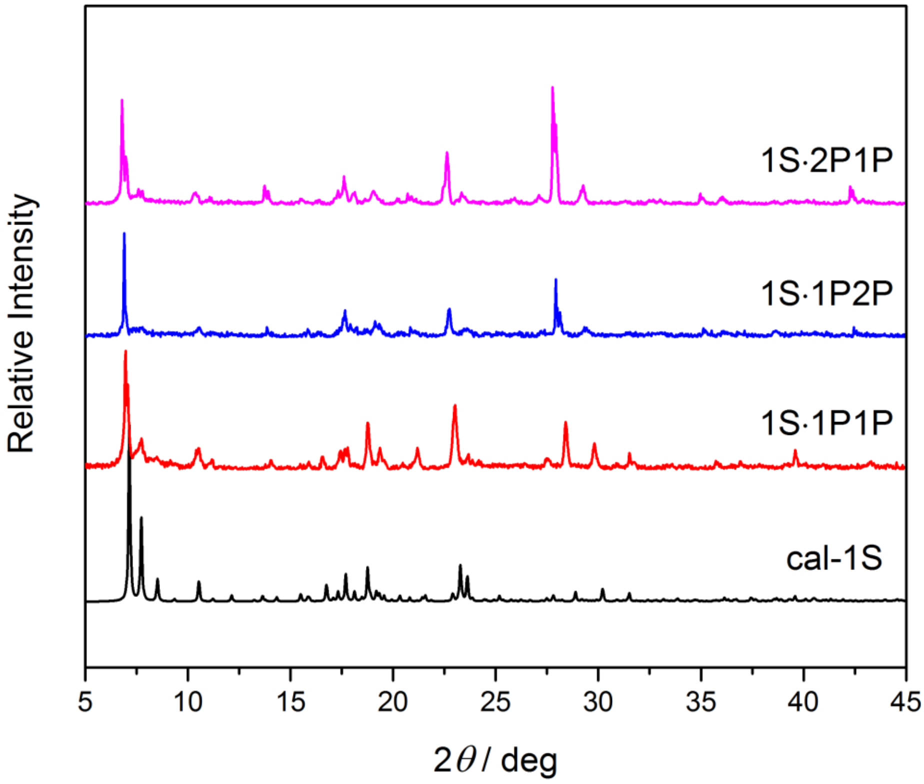

Figure S1. PXRD patterns of calculated **CMOM-1S** (cal-1S), guest molecules encapsulated in **CMOM-1S** with 1P1P, 1P2P and 2P1P (1S·1P1P, 1S·1P2P, 1S·2P1P), respectively.

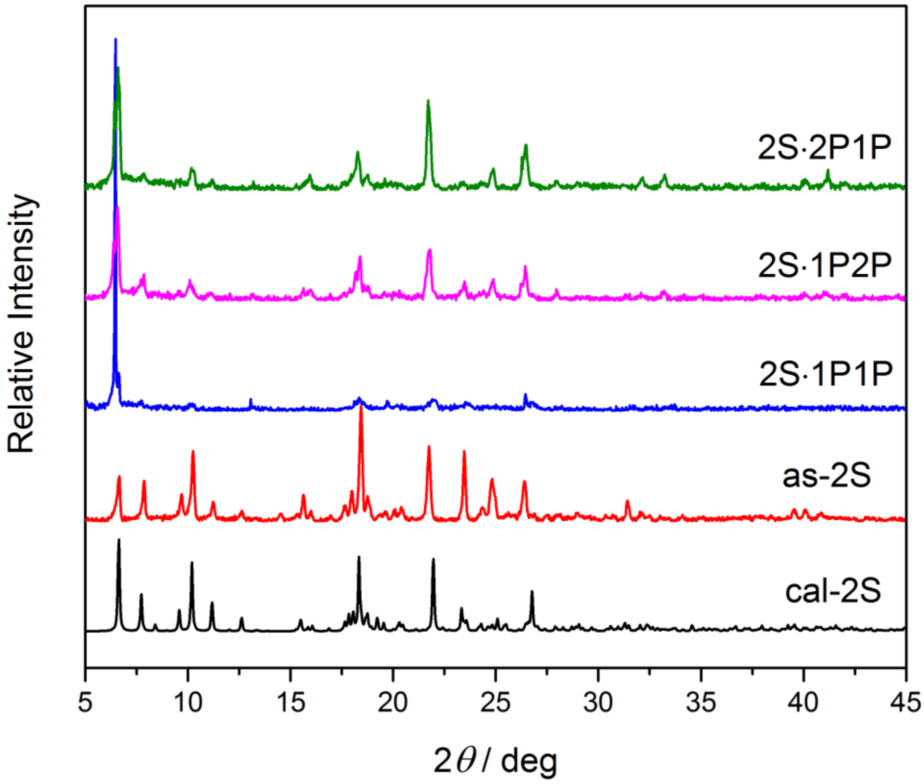

Figure S2. PXRD patterns of calculated **CMOM-2S** (cal-2S), as-synthesized **CMOM-2S** (as-2S), guest molecules encapsulated in **CMOM-2S** with 1P1P, 1P2P and 2P1P (2S·1P1P, 2S·1P2P, 2S·2P1P), respectively.

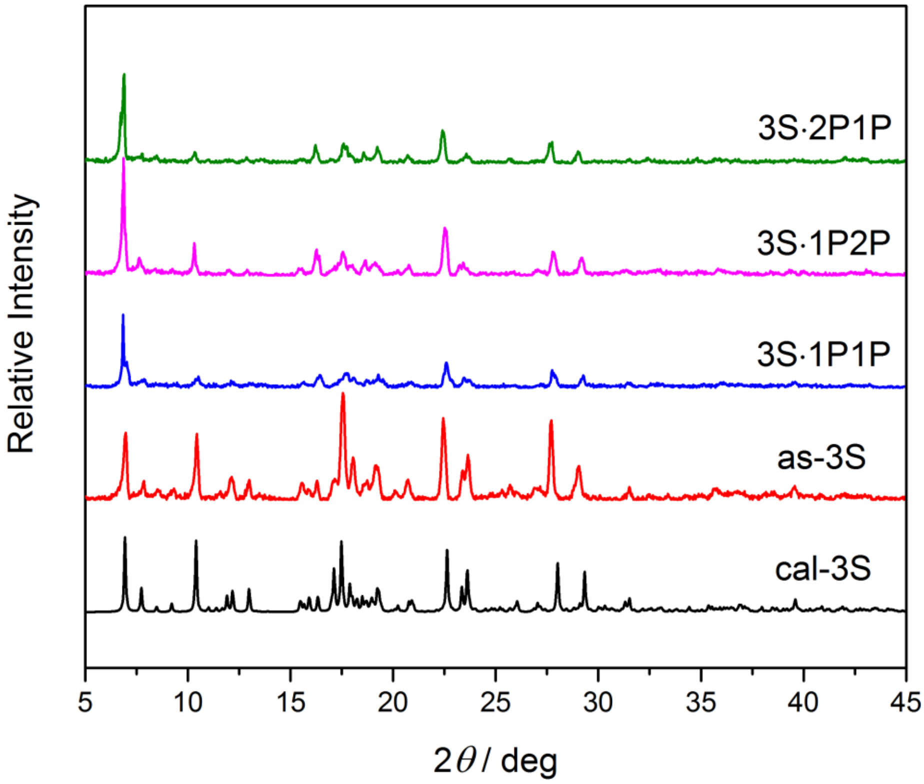

Figure S3. PXRD patterns of calculated **CMOM-3S** (cal-2S), as-synthesized **CMOM-3S** (as-2S), guest molecules encapsulated in **CMOM-3S** with 1P1P, 1P2P and 2P1P (3S·1P1P, 3S·1P2P, 3S·2P1P), respectively.

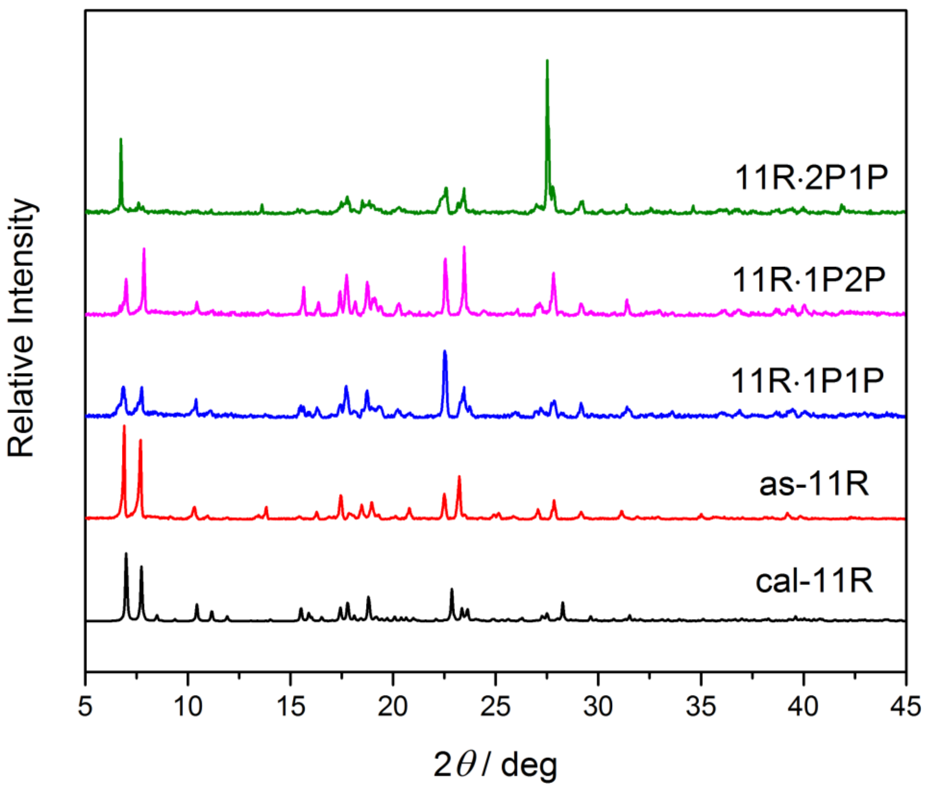

Figure S4. PXRD patterns of calculated **CMOM-11R** (cal-11R), as-synthesized **CMOM-11R** (as-11R), guest molecules encapsulated in **CMOM-11R** with 1P1P, 1P2P and 2P1P (11R·1P1P, 11R·1P2P, 11R·2P1P), respectively.

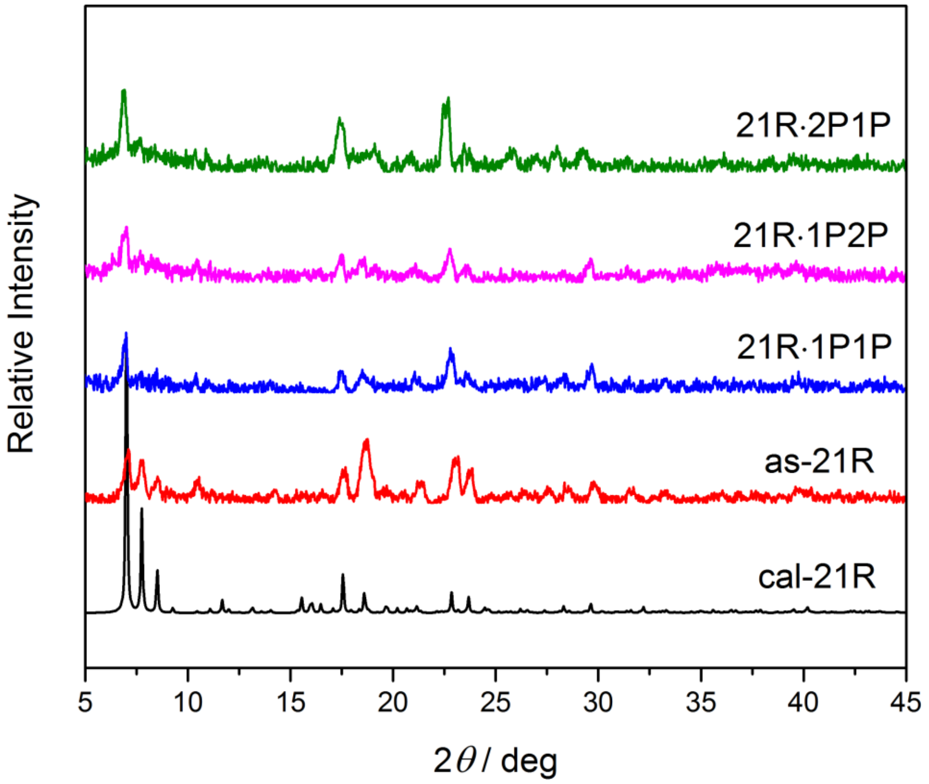

Figure S5. PXRD patterns of calculated **CMOM-21R** (cal-11R), as-synthesized **CMOM-21R** (as-11R), guest molecules encapsulated in **CMOM-21R** with 1P1P, 1P2P and 2P1P (21R·1P1P, 21R·1P2P, 21R·2P1P), respectively.

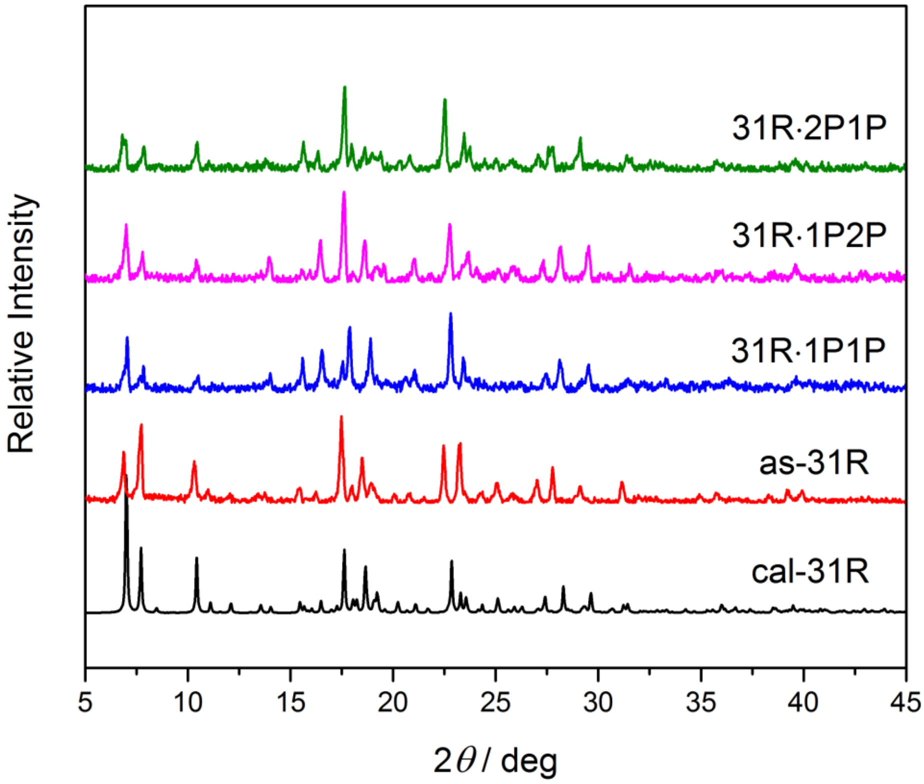

Figure S6. PXRD patterns of calculated **CMOM-31R** (cal-31R), as-synthesized **CMOM-31R** (as-31R), guest molecules encapsulated in **CMOM-31R** with 1P1P, 1P2P and 2P1P (31R·1P1P, 31R·1P2P, 31R·2P1P), respectively.

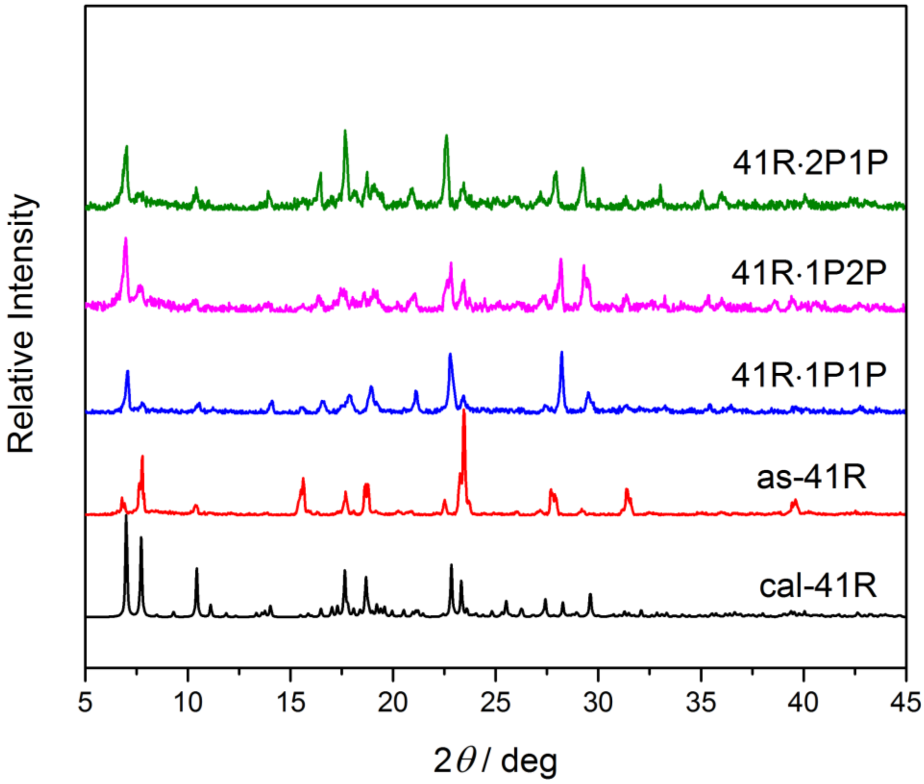

Figure S7. PXRD patterns of calculated **CMOM-41R** (cal-41R), as-synthesized **CMOM-41R** (as-41R), guest molecules encapsulated in **CMOM-41R** with 1P1P, 1P2P and 2P1P (41R·1P1P, 41R·1P2P, 41R·2P1P), respectively.

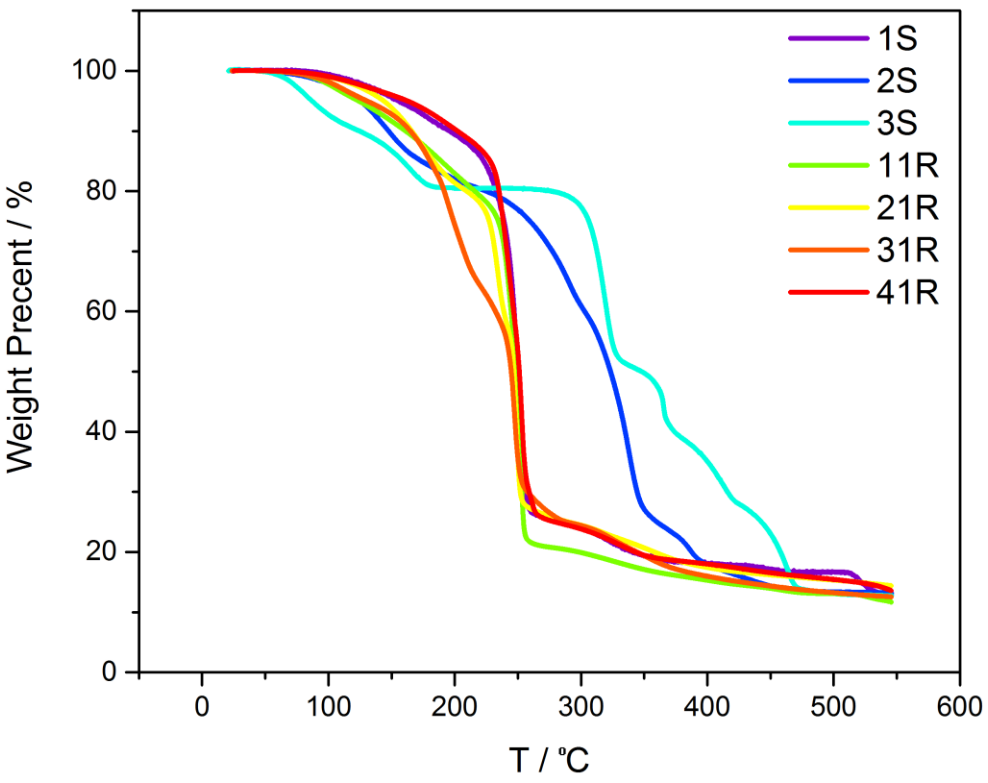

Figure S8. TGA plots of as-synthesized CMOMs.

Additional Figures

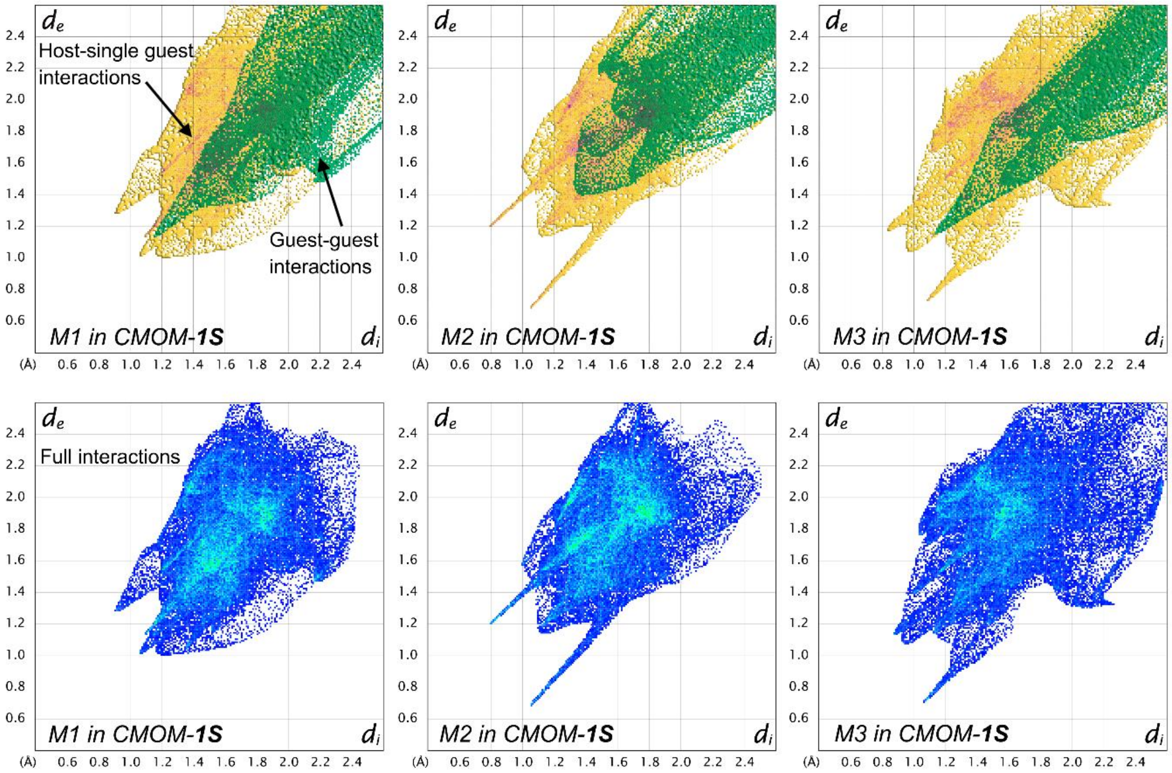

Figure S9. Surface analysis of 1P1P molecules in CMOM-**1S**.

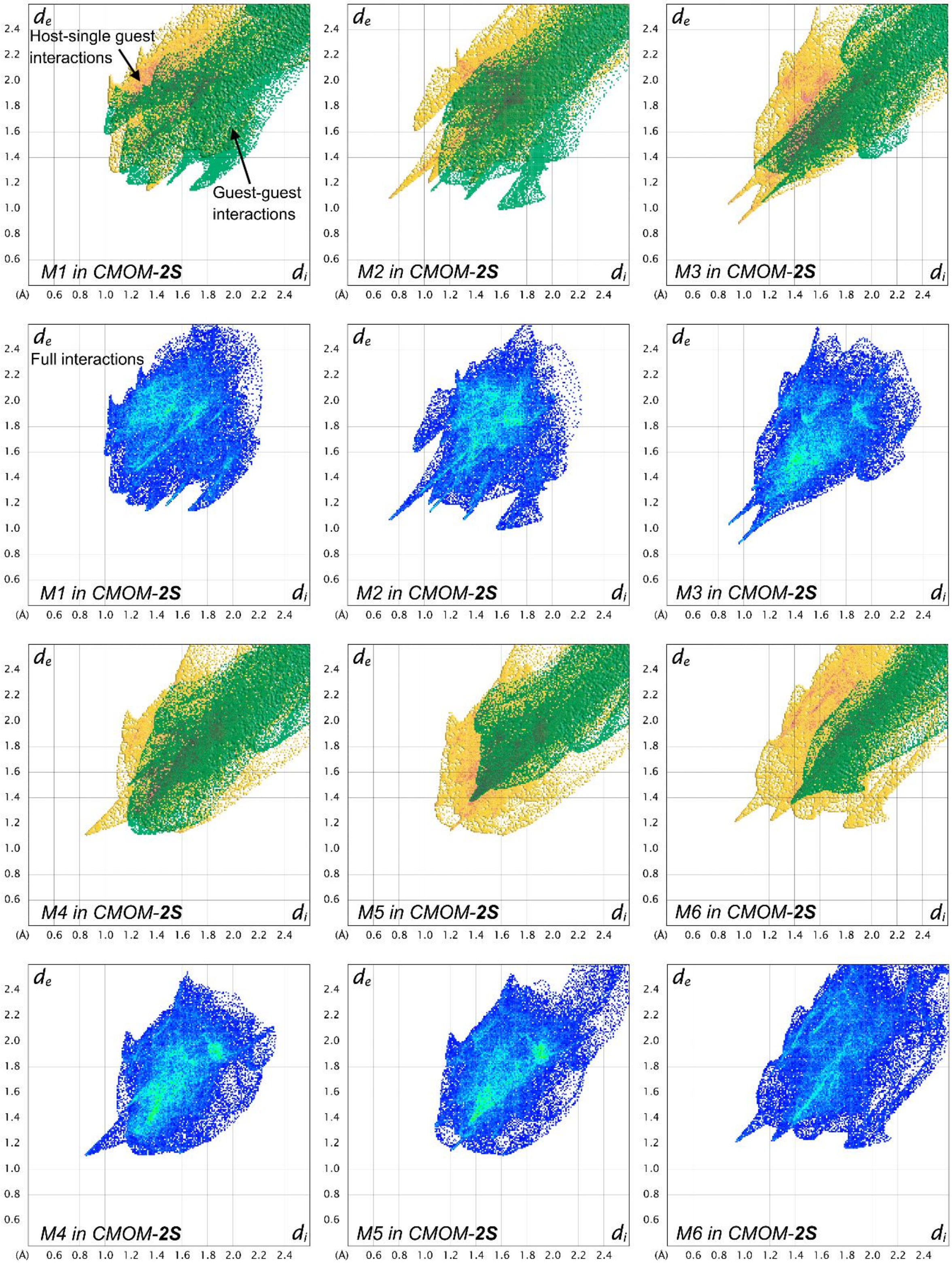

Figure S10. Surface analysis of 1P1P molecules in CMOM-**2S**.

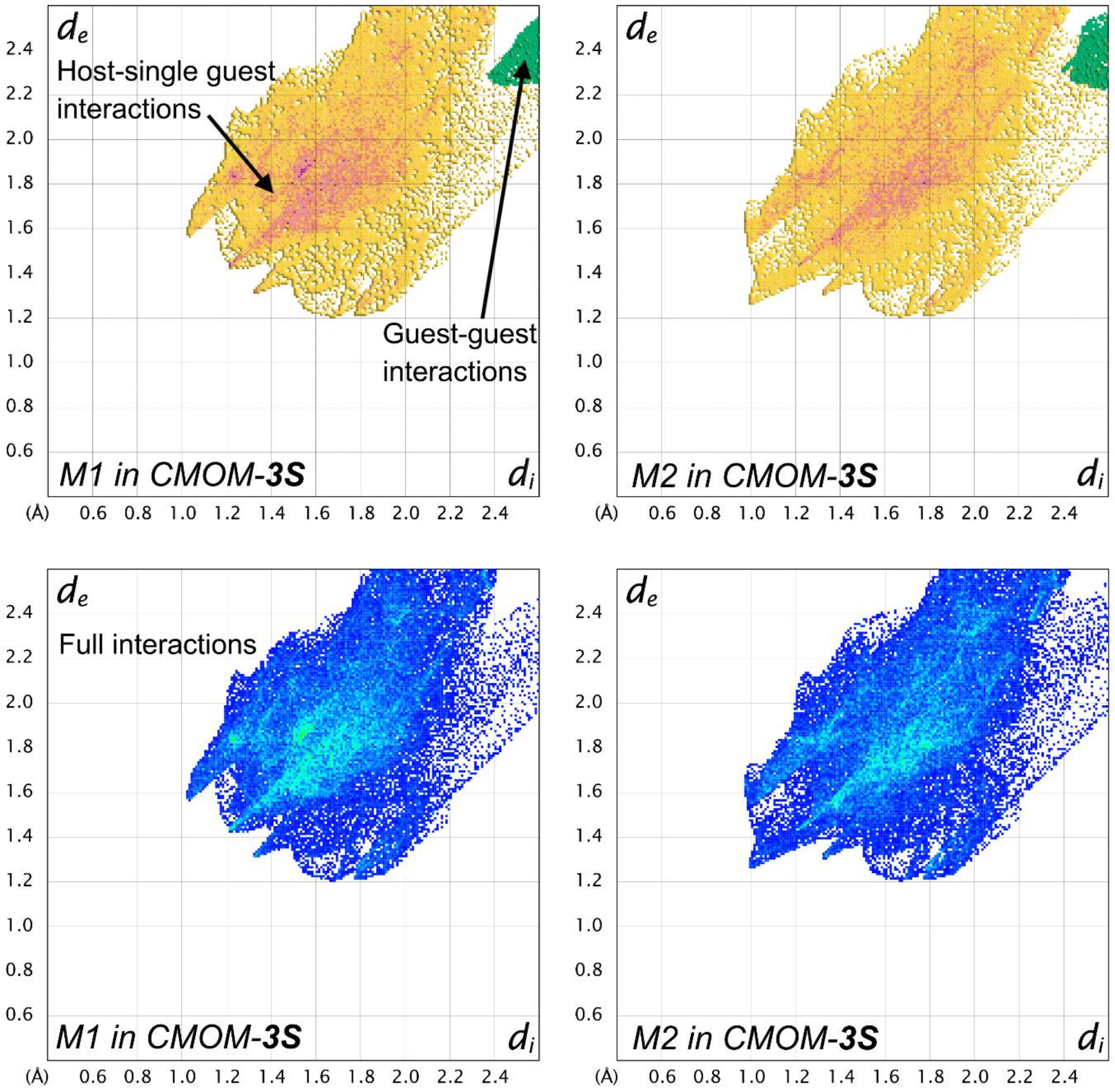

Figure S11. Surface analysis of 1P1P molecules in CMOM-**3S**.

HPLC traces for chiral resolution

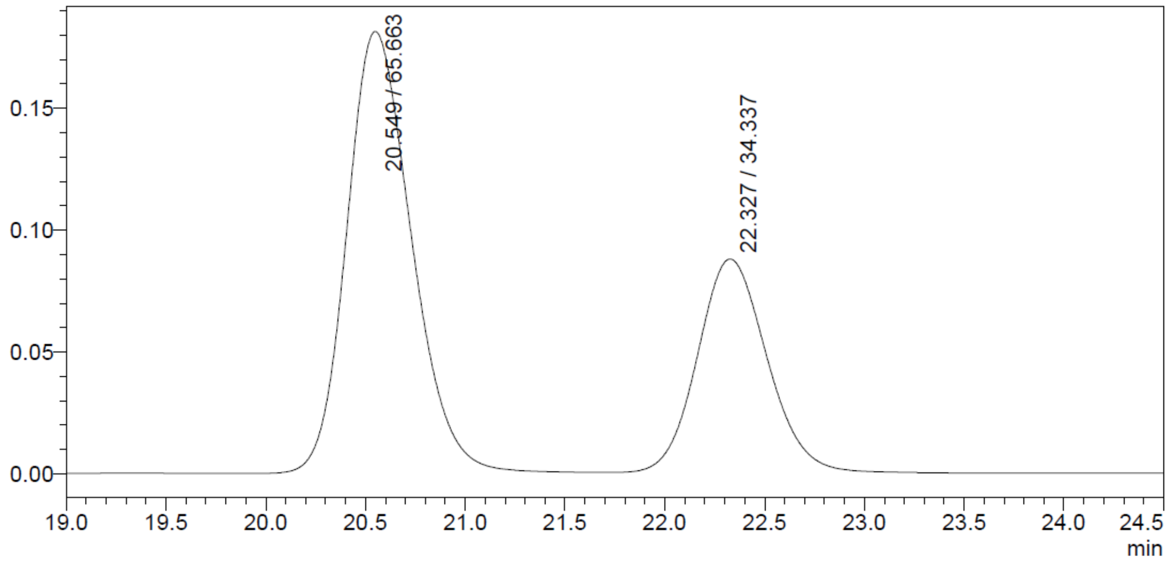

Peak#	Ret. Time	Area	Height	Area%
1	20.549	4097784	181352	65.663
2	22.327	2142825	87887	34.337

Figure S12. HPLC spectra of the solution extracted from 1P1P encapsulated **CMOM-1S** for 5d. Eluent: 99% hexane: 1% isopropanol.

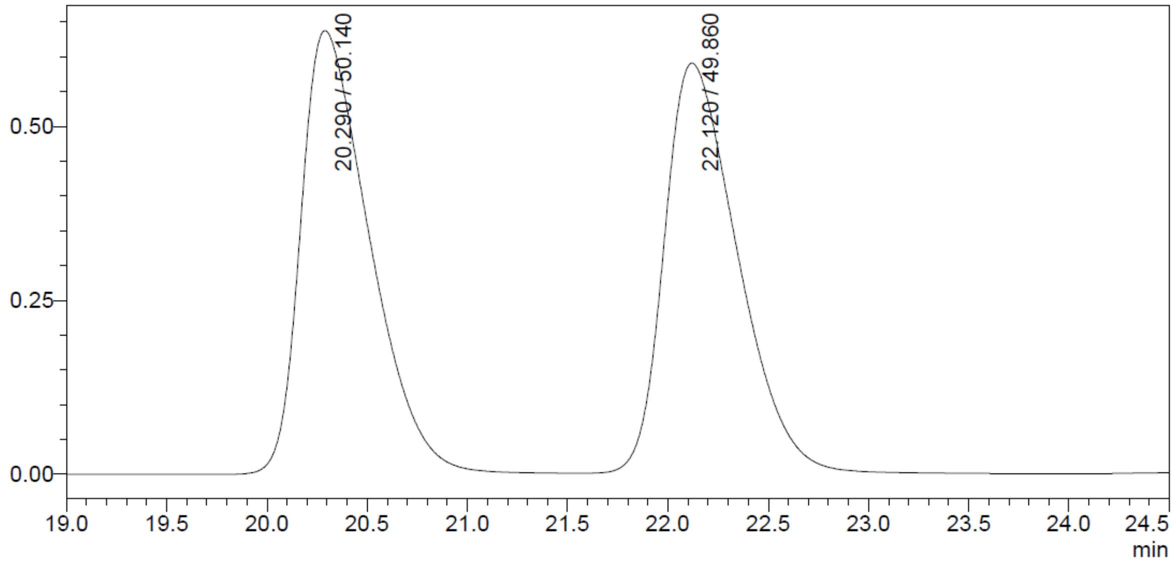

Peak#	Ret. Time	Area	Height	Area%
1	20.290	15239877	637236	50.140
2	22.120	15154683	590313	49.860

Figure S13. HPLC spectra of the solution extracted from 1P1P encapsulated **CMOM-2S** for 5d. Eluent: 99% hexane: 1% isopropanol.

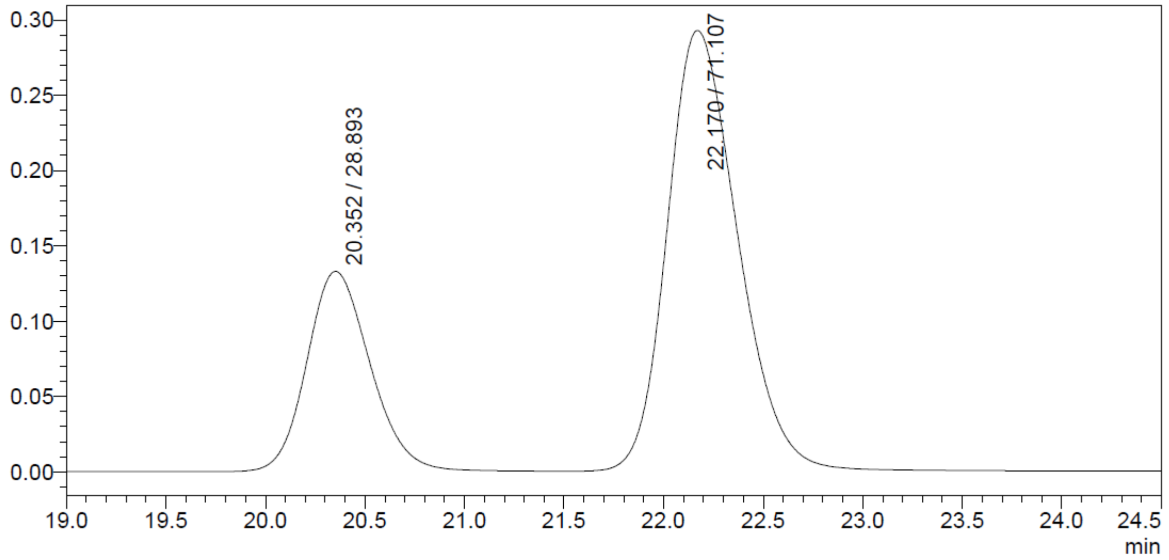

Peak#	Ret. Time	Area	Height	Area%
1	20.352	2947316	132847	28.893
2	22.170	7253376	292831	71.107

Figure S14. HPLC spectra of the solution extracted from 1P1P encapsulated **CMOM-3S** for 5d. Eluent: 99% hexane: 1% isopropanol.

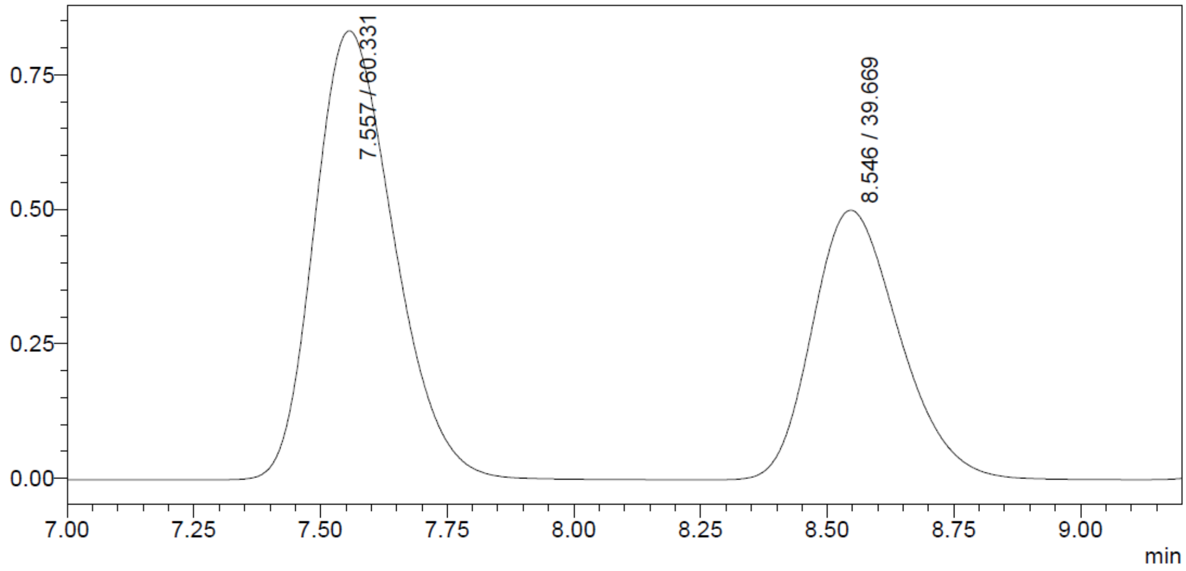

Peak#	Ret. Time	Area	Height	Area%
1	7.557	9124928	833526	60.331
2	8.546	5999918	500448	39.669

Figure S15. HPLC spectra of the solution extracted from 1P2P encapsulated **CMOM-1S** for 5d. Eluent: 95% hexane: 5% isopropanol.

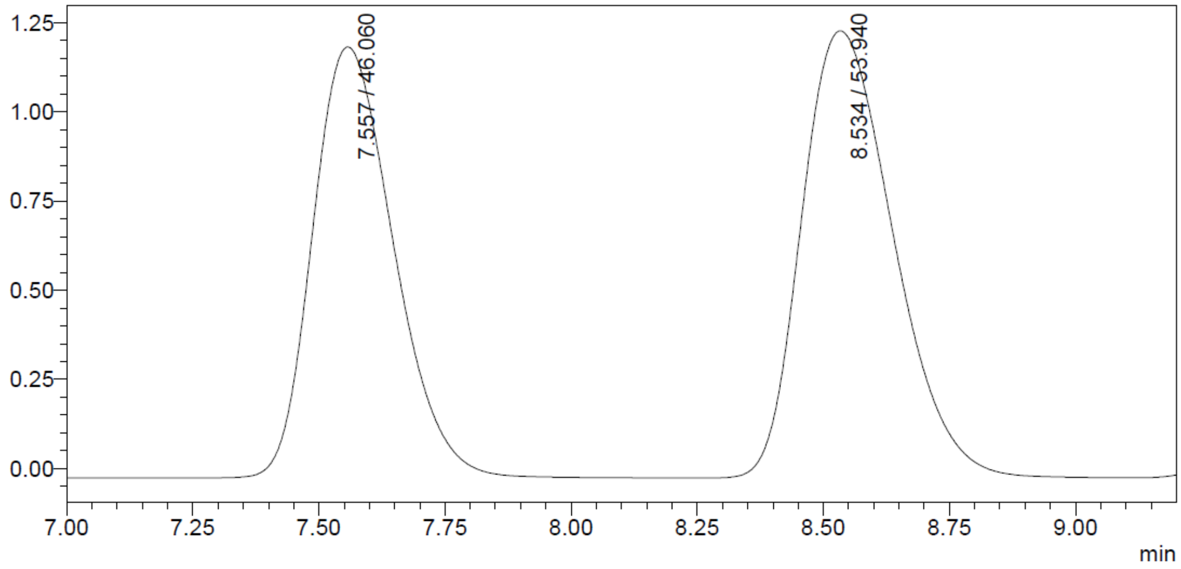

Peak#	Ret. Time	Area	Height	Area%
1	7.557	13480098	1206806	46.060
2	8.534	15786163	1251324	53.940

Figure S16. HPLC spectra of the solution extracted from 1P2P encapsulated **CMOM-2S** for 5d. Eluent: 95% hexane: 5% isopropanol.

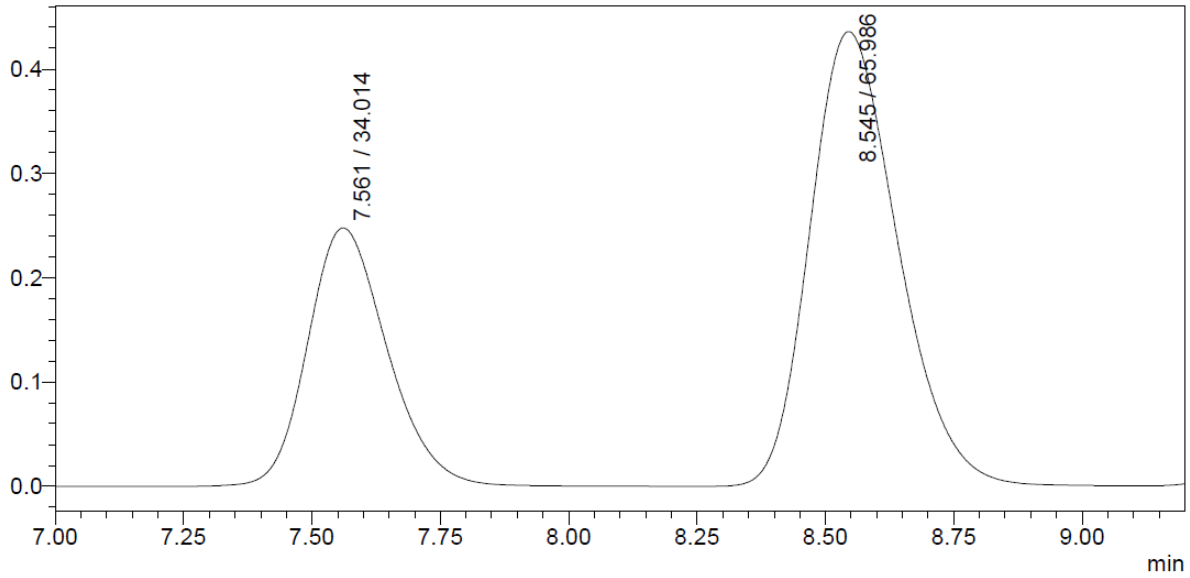

Peak#	Ret. Time	Area	Height	Area%
1	7.561	2674312	247440	34.014
2	8.545	5188002	435523	65.986

Figure S17. HPLC spectra of the solution extracted from 1P2P encapsulated **CMOM-3S** for 5d. Eluent: 95% hexane: 5% isopropanol.

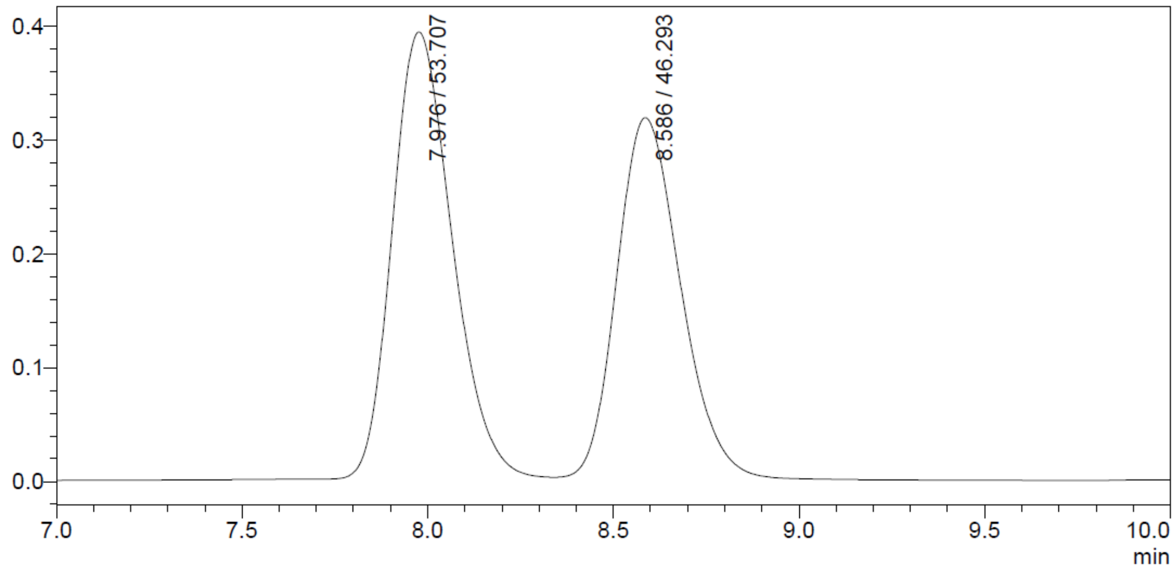

Peak#	Ret. Time	Area	Height	Area%
1	7.976	4478739	393229	53.707
2	8.586	3860458	318535	46.293

Figure S18. HPLC spectra of the solution extracted from 2P1P encapsulated **CMOM-1S** for 5d. Eluent: 95% hexane: 5% isopropanol.

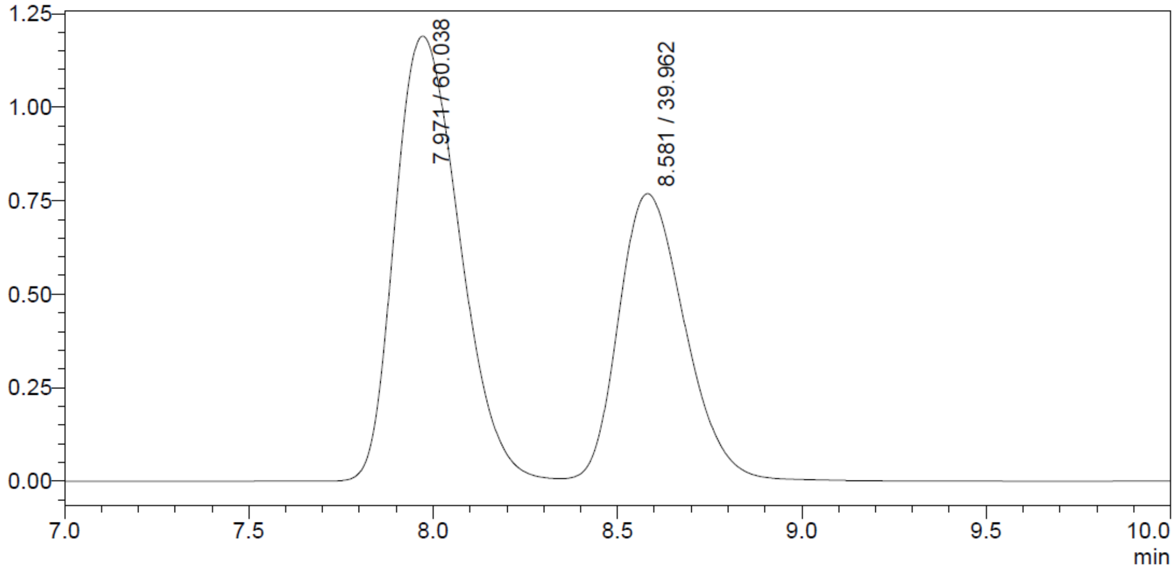

Peak#	Ret. Time	Area	Height	Area%
1	7.971	14513801	1189335	60.038
2	8.581	9660559	767986	39.962

Figure S19. HPLC spectra of the solution extracted from 2P1P encapsulated **CMOM-2S** for 5d. Eluent: 95% hexane: 5% isopropanol.

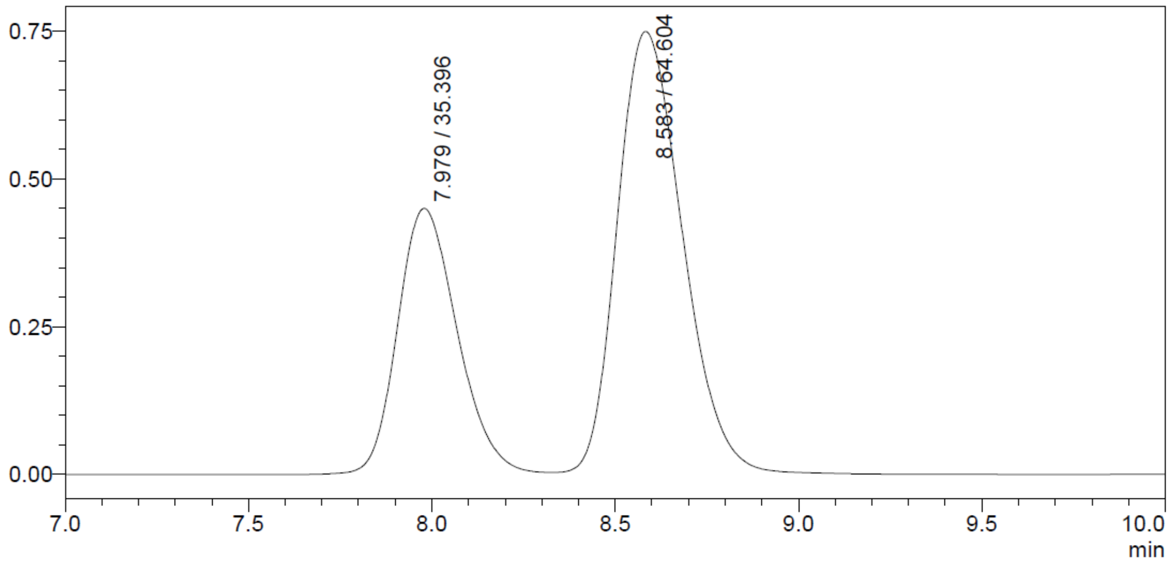

Peak#	Ret. Time	Area	Height	Area%
1	7.979	5131625	450115	35.396
2	8.583	9366281	748880	64.604

Figure S20. HPLC spectra of the solution extracted from 2P1P encapsulated **CMOM-3S** for 5d. Eluent: 95% hexane: 5% isopropanol.

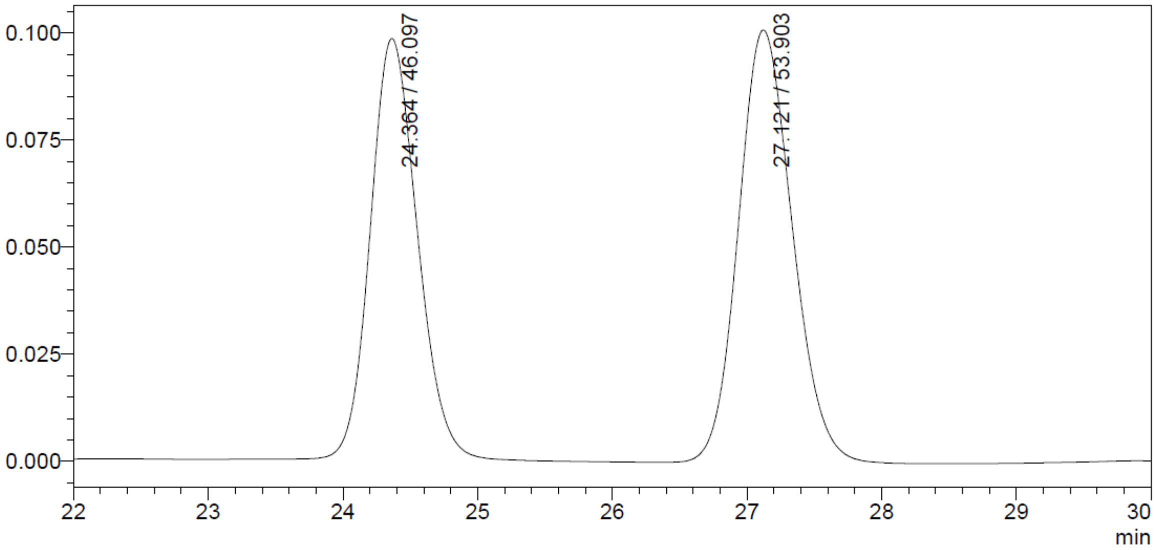

Peak#	Ret. Time	Area	Height	Area%
1	24.364	2405706	98350	46.097
2	27.121	2813092	100978	53.903

Figure S21. HPLC spectra of the solution extracted from 1P1P encapsulated **CMOM-11R** for 5d. Eluent: 99% hexane: 1% isopropanol.

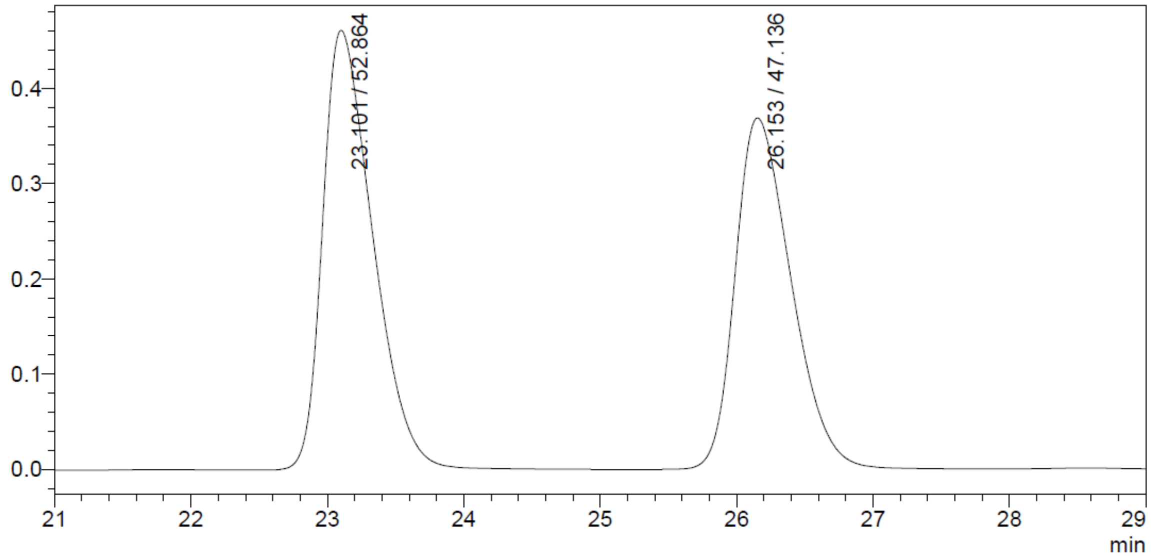

Peak#	Ret. Time	Area	Height	Area%
1	23.101	11792223	461280	52.864
2	26.153	10514690	368788	47.136

Figure S22. HPLC spectra of the solution extracted from 1P1P encapsulated **CMOM-21R** for 5d. Eluent: 99% hexane: 1% isopropanol.

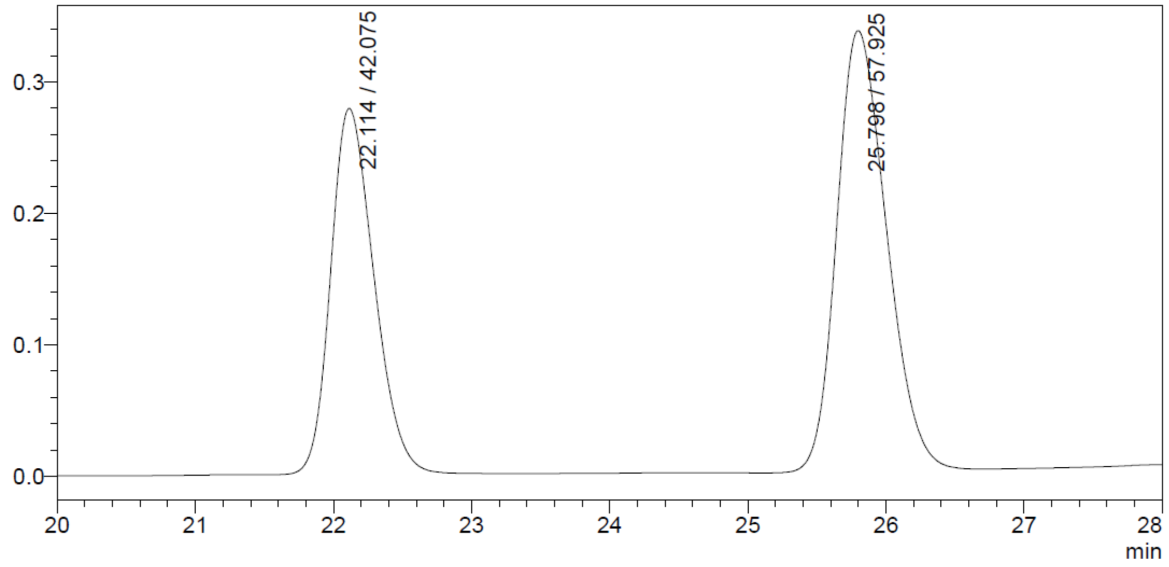

Peak#	Ret. Time	Area	Height	Area%
1	22.114	6099538	278309	42.075
2	25.798	8397141	334885	57.925

Figure S23. HPLC spectra of the solution extracted from 1P1P encapsulated **CMOM-31R** for 5d. Eluent: 99% hexane: 1% isopropanol.

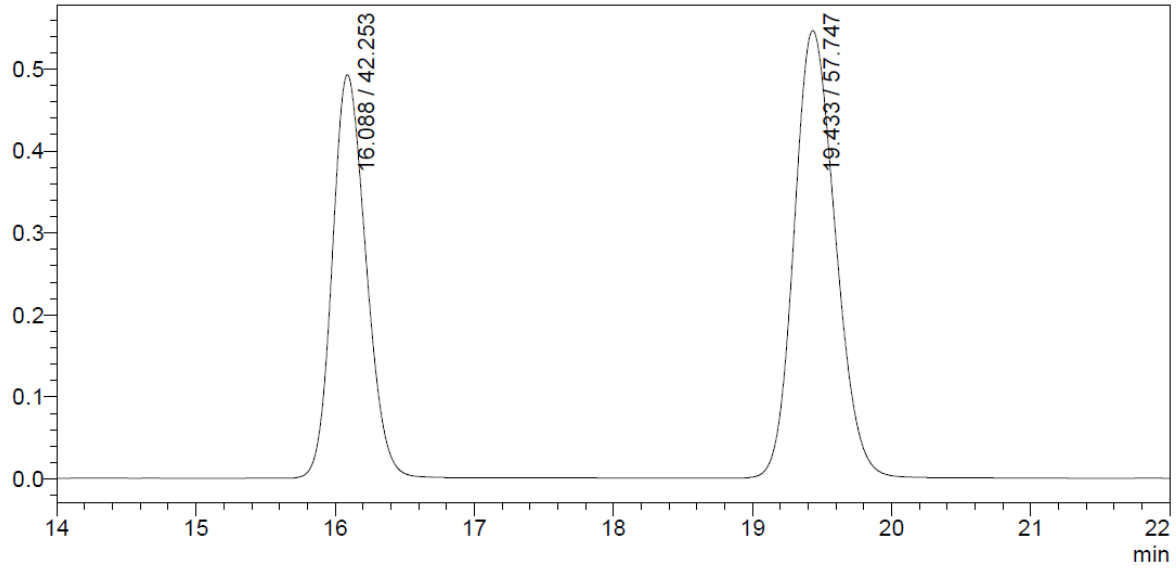

Peak#	Ret. Time	Area	Height	Area%
1	16.088	8301029	492130	42.253
2	19.433	11344841	546232	57.747

Figure S24. HPLC spectra of the solution extracted from 1P1P encapsulated **CMOM-41R** for 5d. Eluent: 99% hexane: 1% isopropanol.

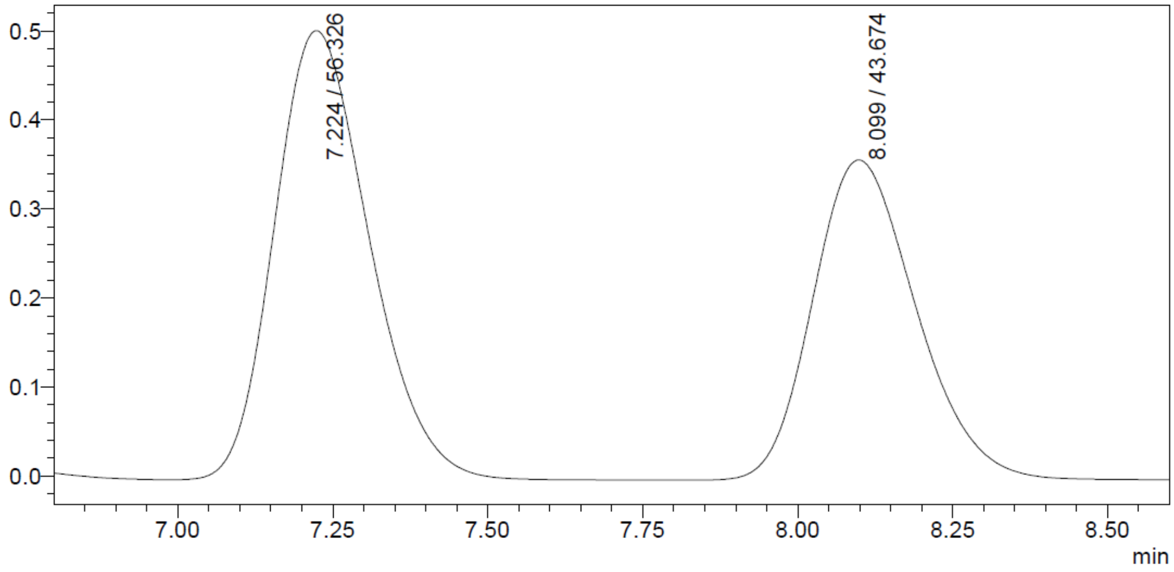

Peak#	Ret. Time	Area	Height	Area%
1	7.224	5365527	503964	56.326
2	8.099	4160320	359105	43.674

Figure S25. HPLC spectra of the solution extracted from 1P2P encapsulated **CMOM-11R** for 5d. Eluent: 95% hexane: 5% isopropanol.

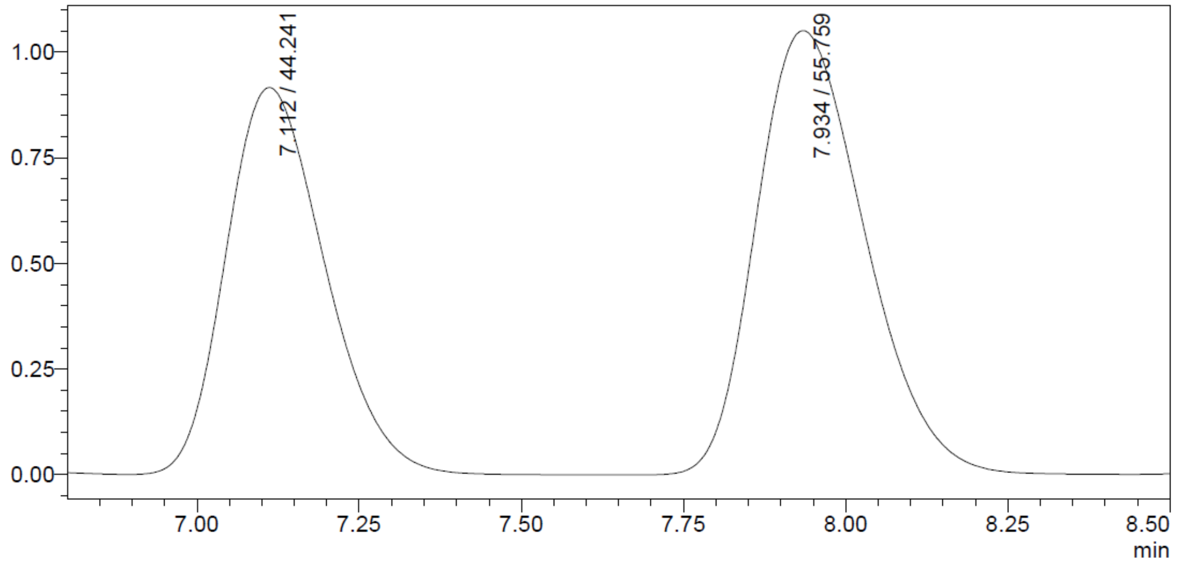

Peak#	Ret. Time	Area	Height	Area%
1	7.112	9705771	914448	44.241
2	7.934	12232770	1048348	55.759

Figure S26. HPLC spectra of the solution extracted from 1P2P encapsulated **CMOM-21R** for 5d. Eluent: 95% hexane: 5% isopropanol.

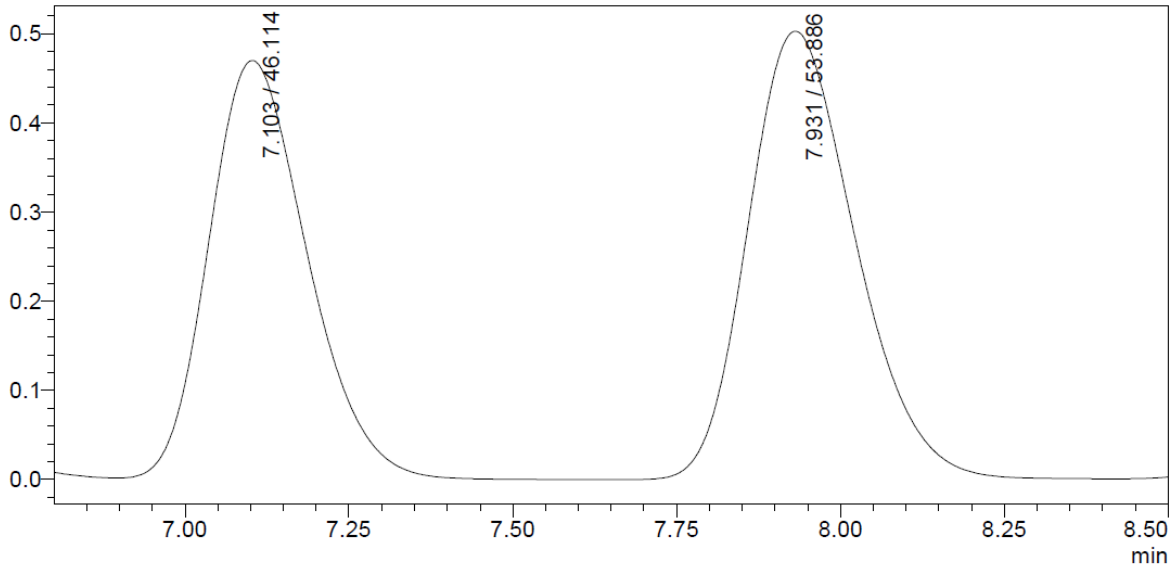

Peak#	Ret. Time	Area	Height	Area%
1	7.103	4878376	468123	46.114
2	7.931	5700586	502242	53.886

Figure S27. HPLC spectra of the solution extracted from 1P2P encapsulated **CMOM-31R** for 5d. Eluent: 95% hexane: 5% isopropanol.

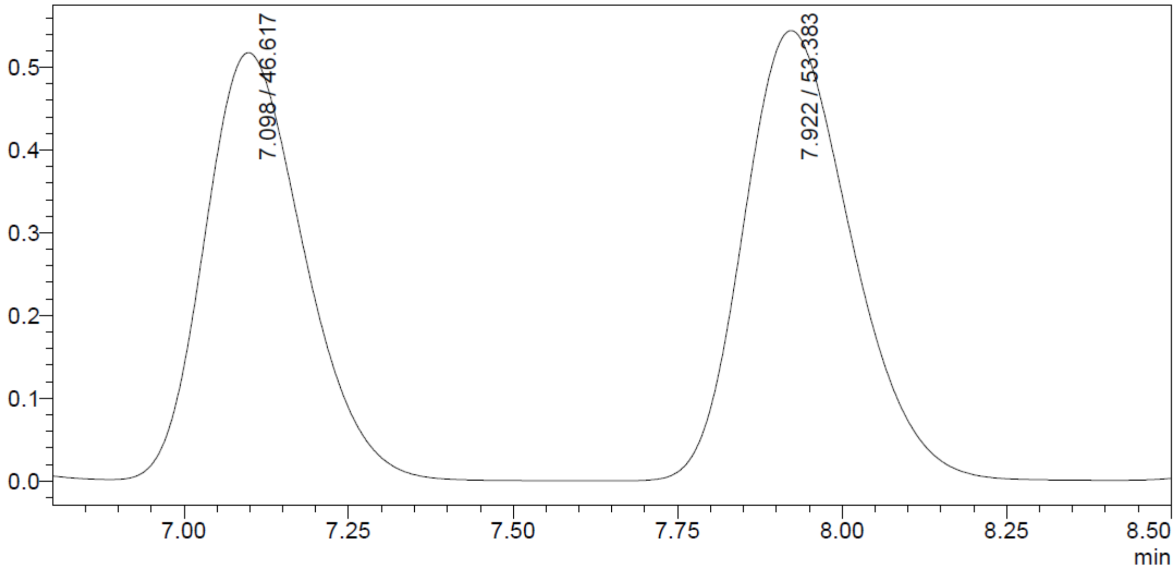

Peak#	Ret. Time	Area	Height	Area%
1	7.098	5404313	516136	46.617
2	7.922	6188657	543689	53.383

Figure S28. HPLC spectra of the solution extracted from 1P2P encapsulated **CMOM-41R** for 5d. Eluent: 95% hexane: 5% isopropanol.

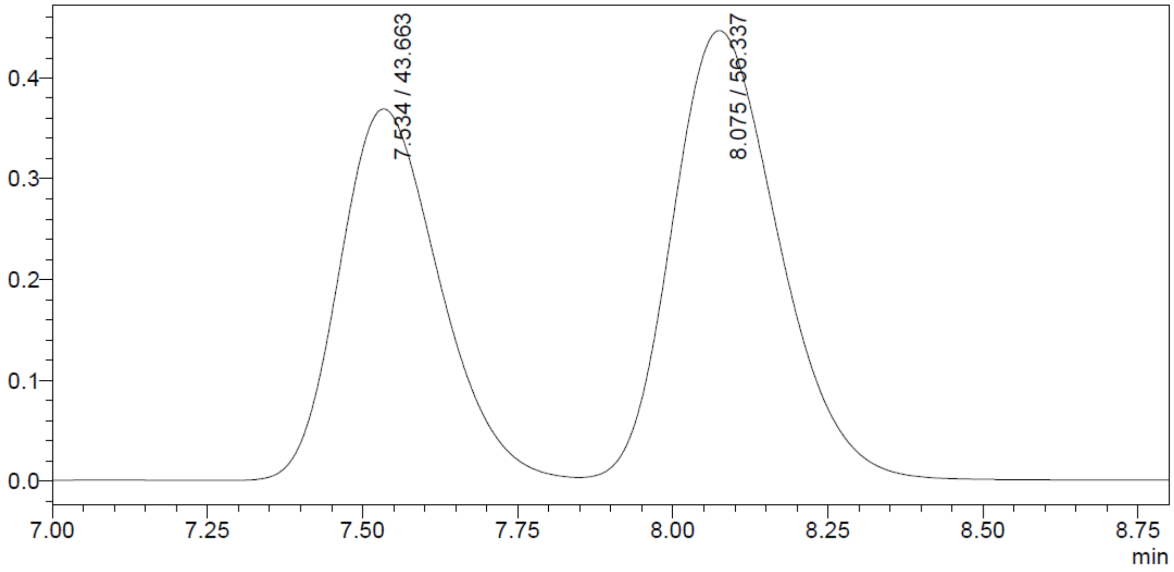

Peak#	Ret. Time	Area	Height	Area%
1	7.534	4126235	367637	43.663
2	8.075	5324003	445464	56.337

Figure S29. HPLC spectra of the solution extracted from 2P1P encapsulated **CMOM-11R** for 5d. Eluent: 95% hexane: 5% isopropanol.

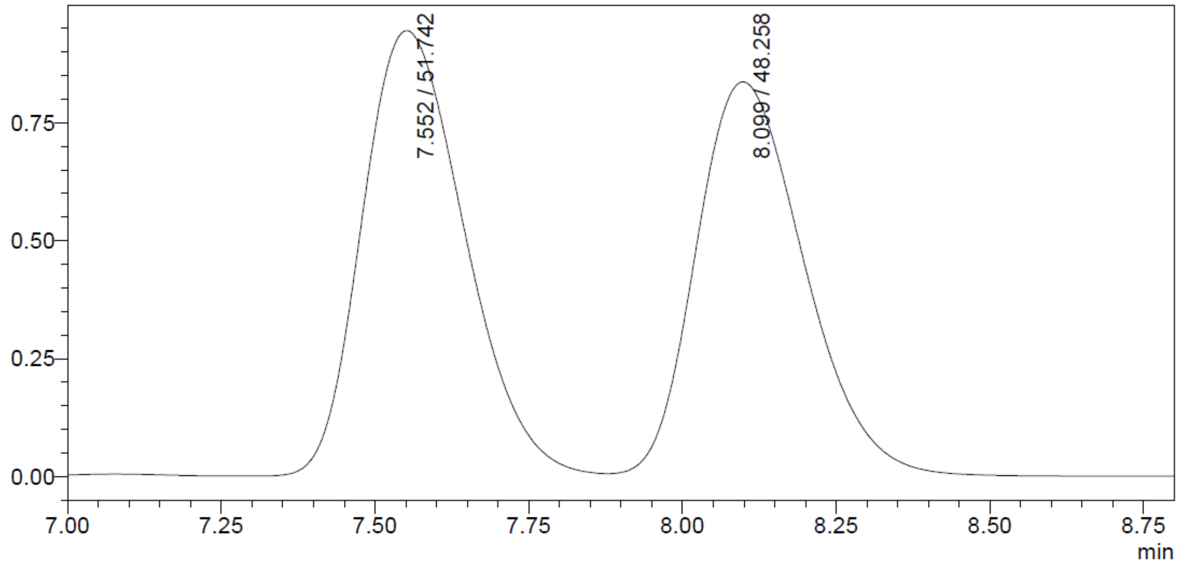

Peak#	Ret. Time	Area	Height	Area%
1	7.552	10947090	943181	51.742
2	8.099	10210094	835315	48.258

Figure S30. HPLC spectra of the solution extracted from 2P1P encapsulated **CMOM-21R** for 5d. Eluent: 95% hexane: 5% isopropanol.

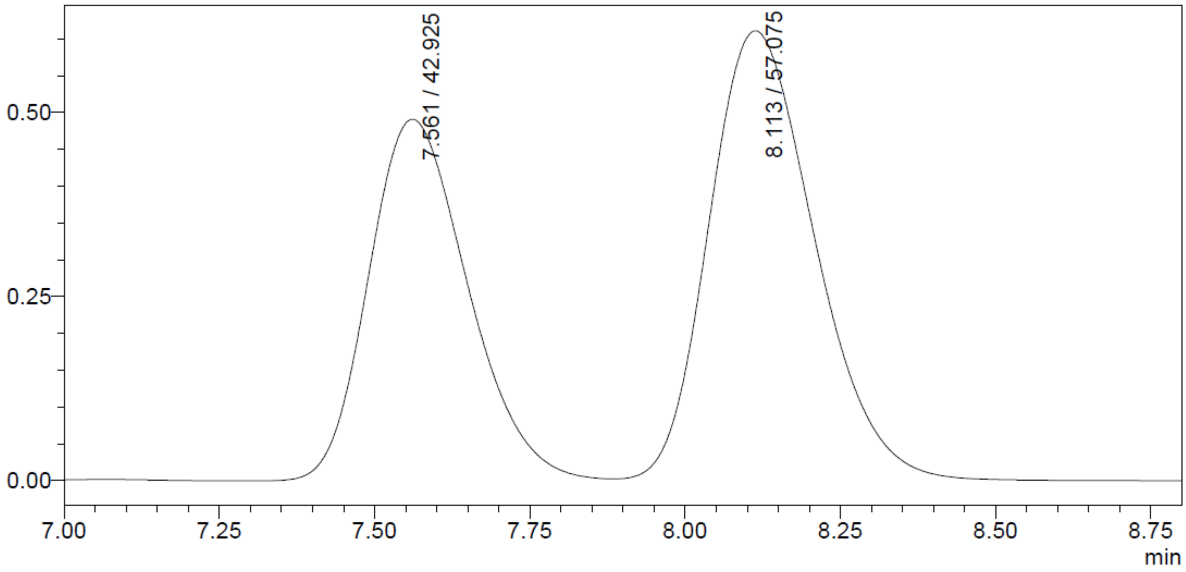

Peak#	Ret. Time	Area	Height	Area%
1	7.561	5450349	490366	42.925
2	8.113	7247148	610638	57.075

Figure S31. HPLC spectra of the solution extracted from 2P1P encapsulated **CMOM-31R** for 5d. Eluent: 95% hexane: 5% isopropanol.

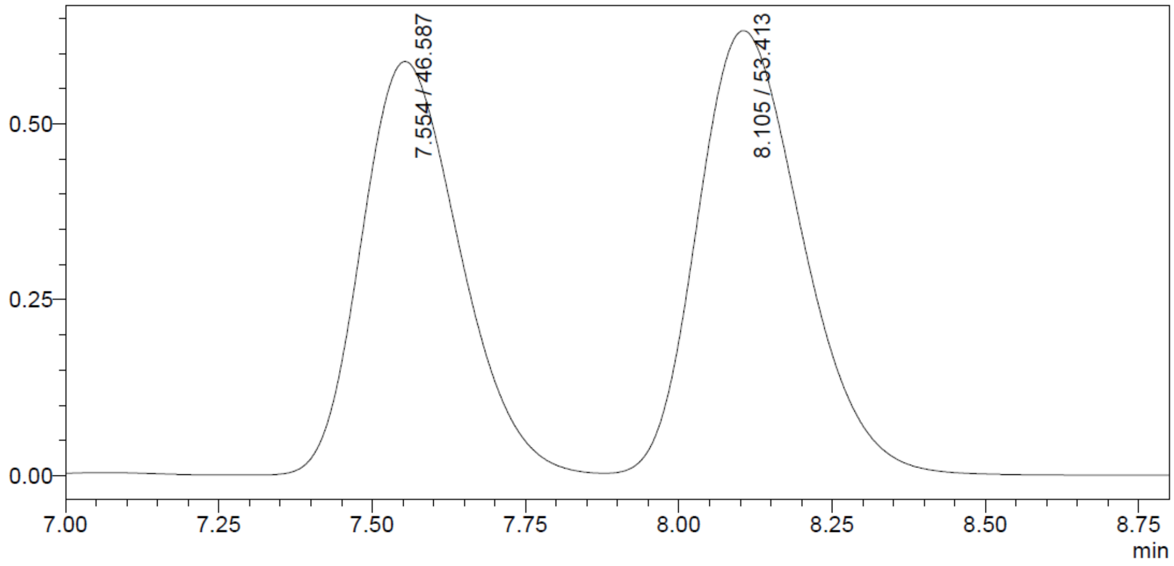

Peak#	Ret. Time	Area	Height	Area%
1	7.554	6569121	587485	46.587
2	8.105	7531511	631545	53.413

Figure S32. HPLC spectra of the solution extracted from 2P1P encapsulated **CMOM-41R** for 5d. Eluent: 95% hexane: 5% isopropanol.

Crystallographic Tables for CMOMs

Table S1. Crystal data and structure refinement for CMOM-2S.

Identification code	CMOM-2S
Empirical formula	$C_{60}H_{48}B_2Co_2F_{14}N_6O_6$
Formula weight	1354.52
Temperature/K	100.3
Crystal system	Monoclinic
Space group	$P2_1$
a/Å	9.6315(3)
b/Å	26.1464(9)
c/Å	11.4211(4)
α/°	90
β/°	94.7122(17)
γ/°	90
Volume/Å^3	2866.44(17)
Z	2
ρ_{calc}g/cm^3	1.569
μ/mm^{-1}	5.452
F(000)	1376.0
2Θ range for data collection/°	6.762 to 155.232
Index ranges	$-9 \leq h \leq 11$, $-33 \leq k \leq 32$, $-14 \leq l \leq 14$
Reflections collected	33897
Independent reflections	10161 [R_{int} = 0.0535, R_{sigma} = 0.0747]
Data/restraints/parameters	10161/231/863
Goodness-of-fit on F^2	1.045
Final R indexes [I>=2σ (I)]	R_1 = 0.0472, wR_2 = 0.1010
Final R indexes [all data]	R_1 = 0.0562, wR_2 = 0.1053
Largest diff. peak/hole / e Å^{-3}	0.56/-0.54
Flack parameter	0.086(4)

Table S2 Crystal data and structure refinement for CMOM-11R.

Identification code	CMOM-11R
Empirical formula	$C_{56.26}H_{42.55}Cl_2Co_2N_{9.71}O_{15.42}$
Formula weight	1290.09
Temperature/K	100.07
Crystal system	Monoclinic
Space group	$P2_1$
a/Å	10.1762(5)
b/Å	25.2353(14)
c/Å	11.4253(7)
α/°	90
β/°	92.392(3)
γ/°	90
Volume/Å^3	2931.5(3)
Z	2
ρ_{calc}g/cm^3	1.462
μ/mm^{-1}	5.917
F(000)	1319.0
2Θ range for data collection/°	7.006 to 130.442
Index ranges	$-11 \le h \le 11$, $-29 \le k \le 29$, $-13 \le l \le 13$
Reflections collected	38004
Independent reflections	9389 [R_{int} = 0.0575, R_{sigma} = 0.0594]
Data/restraints/parameters	9389/128/751
Goodness-of-fit on F^2	1.058
Final R indexes [I>=2σ (I)]	R_1 = 0.0554, wR_2 = 0.1412
Final R indexes [all data]	R_1 = 0.0604, wR_2 = 0.1445
Largest diff. peak/hole / e Å^{-3}	0.58/-0.46
Flack parameter	0.140(4)

Table S3 Crystal data and structure refinement for CMOM-21R.

Identification code	CMOM-21R_sq
Empirical formula	$C_{46}H_{36}Cl_2Co_2N_8O_{12}$
Formula weight	1081.59
Temperature/K	100.05
Crystal system	Monoclinic
Space group	$P2_1$
a/Å	10.3301(4)
b/Å	25.1978(9)
c/Å	11.4221(4)
α/°	90
β/°	94.278(2)
γ/°	90
Volume/Å^3	2964.84(19)
Z	2
ρ_{calc}g/cm^3	1.212
μ/mm^{-1}	5.698
F(000)	1104.0
2Θ range for data collection/°	7.016 to 127.97
Index ranges	-12 ≤ h ≤ 11, -29 ≤ k ≤ 29, -13 ≤ l ≤ 13
Reflections collected	26426
Independent reflections	8890 [R_{int} = 0.0840, R_{sigma} = 0.0973]
Goodness-of-fit on F^2	1.037
Final R indexes [I>=2σ (I)]	R_1 = 0.1255, wR_2 = 0.2803
Final R indexes [all data]	R_1 = 0.1462, wR_2 = 0.2962
Largest diff. peak/hole / e Å^{-3}	1.17/-1.40
Flack parameter	0.323(7)

Table S4. Crystal data and structure refinement for CMOM-31R.

Identification code	CMOM-31R_sq
Empirical formula	$C_{46}H_{36}Cl_2Co_2N_8O_{12}$
Formula weight	1081.59
Temperature/K	100.11
Crystal system	Monoclinic
Space group	$P2_1$
a/Å	10.2648(6)
b/Å	25.2085(14)
c/Å	11.4477(7)
α/°	90
β/°	90.242(4)
γ/°	90
Volume/Å^3	2962.2(3)
Z	2
ρ_{calc}g/cm^3	1.213
μ/mm^{-1}	5.703
F(000)	1104.0
Crystal size/mm^3	0.08 × 0.06 × 0.05
Radiation	CuKα (λ = 1.54178)
2Θ range for data collection/°	7.012 to 123.166
Index ranges	$-11 \leq h \leq 11$, $-28 \leq k \leq 28$, $-12 \leq l \leq 13$
Reflections collected	32897
Independent reflections	9007 [R_{int} = 0.0763, R_{sigma} = 0.0704]
Goodness-of-fit on F^2	1.044
Final R indexes [I>=2σ (I)]	R_1 = 0.1079, wR_2 = 0.2531
Final R indexes [all data]	R_1 = 0.1216, wR_2 = 0.2676
Largest diff. peak/hole / e Å^{-3}	0.83/-0.77
Flack parameter	0.026(9)

Table S5 Crystal data and structure refinement for CMOM-41R.

Identification code	CMOM-41R
Empirical formula	$C_{62.76}H_{53.92}Co_2N_{10.46}O_{16.92}$
Formula weight	1343.22
Temperature/K	99.95
Crystal system	Monoclinic
Space group	$P2_1$
a/Å	10.2576(3)
b/Å	25.2319(8)
c/Å	11.4407(4)
α/°	90
β/°	92.4157(18)
γ/°	90
Volume/Å^3	2958.44(16)
Z	2
ρ_{calc}g/cm^3	1.508
μ/mm^{-1}	5.099
F(000)	1386.0
2Θ range for data collection/°	7.006 to 130.636
Index ranges	$-12 \le h \le 12$, $-29 \le k \le 29$, $-13 \le l \le 13$
Reflections collected	38384
Independent reflections	9300 [R_{int} = 0.0443, R_{sigma} = 0.0548]
Data/restraints/parameters	9300/585/1027
Goodness-of-fit on F^2	1.054
Final R indexes [I>=2σ (I)]	R_1 = 0.0413, wR_2 = 0.0905
Final R indexes [all data]	R_1 = 0.0516, wR_2 = 0.0954
Largest diff. peak/hole / e Å^{-3}	0.71/-0.47
Flack parameter	0.066(4)

Table S6. Crystal data and structure refinement for 2S·1P1P.

Identification code	2S·1P1P
Empirical formula	$C_{118.24}H_{108.99}B_4Co_4F_{16}N_{12}O_{14.91}$
Formula weight	2519.70
Temperature/K	99.89
Crystal system	Monoclinic
Space group	$P2_1$
a/Å	11.4090(4)
b/Å	26.5297(9)
c/Å	19.5485(7)
α/°	90
β/°	93.1780(15)
γ/°	90
Volume/Å^3	5907.8(4)
Z	2
ρ_{calc}g/cm^3	1.416
μ/mm^{-1}	5.113
F(000)	2588.0
2Θ range for data collection/°	4.528 to 133.666
Index ranges	$-12 \le h \le 13$, $-31 \le k \le 31$, $-23 \le l \le 23$
Reflections collected	70522
Independent reflections	19667 [R_{int} = 0.0725, R_{sigma} = 0.0802]
Goodness-of-fit on F^2	1.043
Final R indexes [I>=2σ (I)]	R_1 = 0.0756, wR_2 = 0.1622
Final R indexes [all data]	R_1 = 0.0968, wR_2 = 0.1750
Largest diff. peak/hole / e Å^{-3}	0.97/-0.68
Flack parameter	0.206(4)

Table S7. Crystal data and structure refinement for 3S·2P1P.

Identification code	3S·2P1P
Empirical formula	$C_{55.99}H_{48.07}Co_2F_6N_6O_{12.9}S_2$
Formula weight	1307.25
Temperature/K	100.26
Crystal system	Monoclinic
Space group	$P2_1$
a/Å	10.2309(3)
b/Å	25.4904(8)
c/Å	11.4258(4)
α/°	90
β/°	91.6204(19)
γ/°	90
Volume/Å^3	2978.54(17)
Z	2
ρ_{calc}g/cm^3	1.458
μ/mm^{-1}	5.758
F(000)	1338.0
2Θ range for data collection/°	6.936 to 118.19
Index ranges	-11 ≤ h ≤ 11, -28 ≤ k ≤ 28, -11 ≤ l ≤ 12
Reflections collected	28768
Independent reflections	8159 [R_{int} = 0.0632, R_{sigma} = 0.0773]
Goodness-of-fit on F^2	1.063
Final R indexes [I>=2σ (I)]	R_1 = 0.0694, wR_2 = 0.1558
Final R indexes [all data]	R_1 = 0.0881, wR_2 = 0.1661
Largest diff. peak/hole / e Å^{-3}	0.59/-0.55
Flack parameter	0.118(5)

Reference

(1) S. Y. Zhang, L. Wojtas, M. J. Zaworotko, *J. Am. Chem. Soc.*, **2015**, 137, 12045.

(2) S. Y. Zhang, C. X. Yang, W. Shi, X. P. Yan, P. Cheng, L. Wojtas, M. J. Zaworotko, *Chem*, **2017**, 3, 281.

(3) Bruker, APEX2, **2010**, Bruker AXS Inc., Madison, Wisconsin, USA.

(4) Bruker, SAINT, **2009**, Data Reduction Software, Bruker AXS Inc., Madison, Wisconsin, USA.

(5) Sheldrick, G. M. SADABS, **2008**, University of Gottingen, Germany.

(6) Sheldrick, G.M. SHELXL-97, **1997**, Program for the Refinement of Crystal.

(7) Farrugia, L. J. *J. Appl. Cryst*. **2012**, 45, 849.

(8) Dolomanov, O.V., Bourhis, L.J., Gildea, R.J, Howard, J.A.K., Puschmann, H. *J. Appl. Cryst*. **2009**, 42, 339.

(9) Spek, A. L. *Acta Crystallogr., Sect. D: Biol. Crystallogr*. **2009**, 65, 148.

(10) Wolff, S. K.; Grimwood, D. J.; McKinnon, J. J.; Jayatilaka, D.; Spackman, M. A. CrystalExplorer 1.5; University of Western Australia, Perth, Australia, 2006
